# Is Glacial Meltwater a Secondary Source of Legacy Contaminants to Arctic Coastal Food Webs?

**DOI:** 10.1021/acs.est.1c07062

**Published:** 2022-04-26

**Authors:** Maeve McGovern, Nicholas A. Warner, Katrine Borgå, Anita Evenset, Pernilla Carlsson, Emelie Skogsberg, Janne E. Søreide, Anders Ruus, Guttorm Christensen, Amanda E. Poste

**Affiliations:** † Norwegian Institute for Water Research, Tromsø 9007, Norway; ‡ Department of Arctic Marine Biology, UiT, The Arctic University of Norway, Tromsø 9019, Norway; § University Centre on Svalbard, Longyearbyen 9170, Norway; ∥ The Fram Centre, NILU-Norwegian Institute for Air Research, Tromsø 9007, Norway; ⊥ Department of Chemistry, UiT, The Arctic University of Norway, Tromsø 9019, Norway; # Thermo Fischer Scientific, Bremen 28199, Germany; ¶ Department of Biosciences, University of Oslo, Oslo 0316, Norway; ∇ Centre for Biogeochemistry in the Anthropocene (CBA), University of Oslo, Oslo 0316, Norway; ○ Akvaplan-niva, Fram Centre, Tromsø 9007, Norway; ⧫ Faculty of Environmental Sciences and Natural Resource Management, Norwegian University of Life Sciences, Ås 1430, Norway; †† Norwegian Institute for Water Research, Oslo 0579, Norway

**Keywords:** climate change, persistent organic pollutants, chiral pesticides, zooplankton, zoobenthos, sculpin, stable isotopes, Svalbard

## Abstract

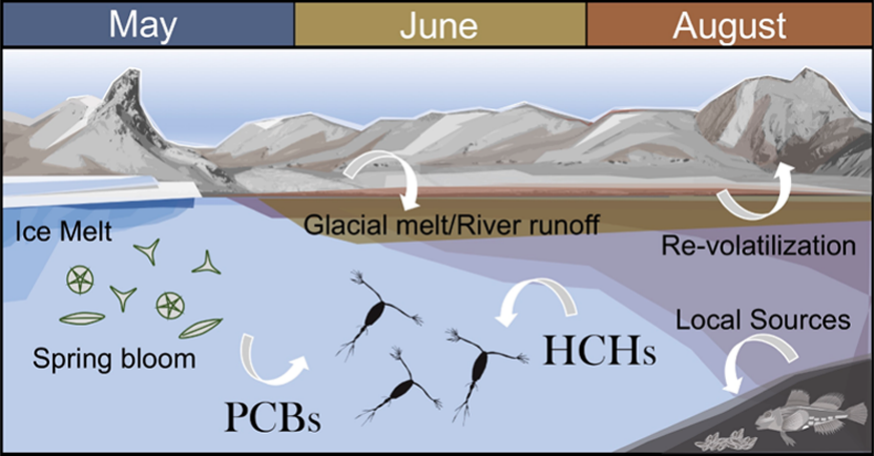

Climate change-driven increases in air and sea temperatures are rapidly thawing the Arctic cryosphere with potential for remobilization and accumulation of legacy persistent organic pollutants (POPs) in adjacent coastal food webs. Here, we present concentrations of selected POPs in zooplankton (spatially and seasonally), as well as zoobenthos and sculpin (spatially) from Isfjorden, Svalbard. Herbivorous zooplankton contaminant concentrations were highest in May [e.g., ∑polychlorinated biphenyls (_8_PCB); 4.43, 95% CI: 2.72–6.3 ng/g lipid weight], coinciding with the final stages of the spring phytoplankton bloom, and lowest in August (∑_8_PCB; 1.6, 95% CI: 1.29–1.92 ng/g lipid weight) when zooplankton lipid content was highest, and the fjord was heavily impacted by sediment-laden terrestrial inputs. Slightly increasing concentrations of α-hexachlorocyclohexane (α-HCH) in zooplankton from June (1.18, 95% CI: 1.06–1.29 ng/g lipid weight) to August (1.57, 95% CI: 1.44–1.71 ng/g lipid weight), alongside a higher percentage of α-HCH enantiomeric fractions closer to racemic ranges, indicate that glacial meltwater is a secondary source of α-HCH to fjord zooplankton in late summer. Except for α-HCH, terrestrial inputs were generally associated with reduced POP concentrations in zooplankton, suggesting that increased glacial melt is not likely to significantly increase exposure of legacy POPs in coastal fauna.

## Abstract 2

Glacial meltwater is not an important secondary source of legacy contaminants to coastal zooplankton and zoobenthos in Isfjorden, Svalbard.

## 1 Introduction

The Arctic cryosphere is melting at an unprecedented rate,^1,2^ yet little information exists on the potential role of melting glaciers and thawing permafrost as secondary sources of legacy contaminants to coastal food webs. In Svalbard, annual runoff has increased more than 35% since 1980, mainly due to enhanced glacial melt and transferring high quantities of meltwater to coastal areas.^3,4^ Glaciers, snow caps, and Arctic tundra contain stores of contaminants,^5^ including persistent organic pollutants (POPs), that have been atmospherically transported from lower latitudes^6^ and deposited on the Arctic environment.^7−10^ Runoff from these systems potentially represents a secondary source of legacy contaminants, including hexachlorobenzene (HCB), polychlorinated biphenyls (PCBs), dichlorodiphenyltrichloroethane (DDTs), hexachlorocyclohexane (HCHs), and chlordane pesticides, to the coastal zone.^11−15^

In addition to remobilization of these legacy POPs, climate change-driven impacts on biogeochemistry and ecology are likely to have implications for the accumulation and trophic transfer of contaminants to the coastal environment.^2,16−19^ Increased temperatures and diminished sea ice may lead to enhanced volatilization of POPs across the air–water interface, resulting in reduced dissolved concentrations available for uptake.^20^ Phytoplankton and high biomass events, like the spring bloom, can facilitate the uptake of dissolved POPs into the food web or their removal from the water column.^21^ Similarly, the high load of suspended particles associated with riverine and glacial runoff on Svalbard^22^ may effectively remove POPs with high particle affinity from the water column.^23^ Furthermore, shifts in carbon source and food web structure can lead to changes in contaminant pathways in marine food webs.^24^ Recent studies suggest that terrestrially derived organic matter may provide an additional energy source to littoral amphipods and marine zooplankton in Isfjorden, Svalbard, during the melt season.^25,26^ Such terrestrial carbon utilization could alter exposure and potential trophic transfer of POPs to coastal ecosystems. Many of these expected changes also occur seasonally in the Isfjorden system, with sea ice present from December to May, presenting the opportunity to investigate these physical and ecological impacts on contaminant dynamics. Given the potential for climate-driven increases in inputs of POPs from secondary sources,^27^ it is important to elucidate the various biogeochemical and ecological processes affecting accumulation and trophic transfer of POPs in the seasonally dynamic coastal zone in the high Arctic in order to assess the potential for increased contamination of coastal food webs.

Chiral compounds exist as enantiomers that have the same physical–chemical properties but can display different affinity/interaction with biological molecules (e.g., enzymes). These differences can give rise to enantiomer enrichment through biological enantiomer-selective processes.^28,29^ Enantiomeric fractions (EFs) of chiral pesticides allow for relative differentiation between fresh and degraded sources of contaminants and receiving marine systems.^30^ Previous studies have used EFs in Svalbard zooplankton^31,32^ to distinguish contaminant sources in relation to ice melt, water mass transport, and biological processes in the water column (e.g., spring bloom).

In the present study, we target several POP groups, covering a broad range of physicochemical properties together with isomeric and enantioselective analysis.^31,33^ We pair these results with environmental data and stable isotope analysis of carbon (for assessing carbon source) and nitrogen (trophic position) to determine the relative importance of terrestrial runoff to contaminant loads in coastal fauna in Isfjorden, Svalbard. Zooplankton, which drift with water masses and represent a key link between the base of the food web and higher trophic levels, were chosen to reflect seasonal variations in contamination, while the more stationary benthic invertebrates and sculpin were selected to study temporally integrated spatial differences among the sampled fjord arms. For zooplankton, we targeted three key time points in the High Arctic summer: the spring bloom in May, the snowmelt period in June, and late-summer glacial melt in August. Through examination of contaminant dynamics together with spatial and seasonal physical and ecological processes, we aim to gain a better understanding of contaminant sources and pathways in the dynamic High Arctic coastal zone.

## 2 Methods

### 2.1 Field Sampling

**Figure 1:**
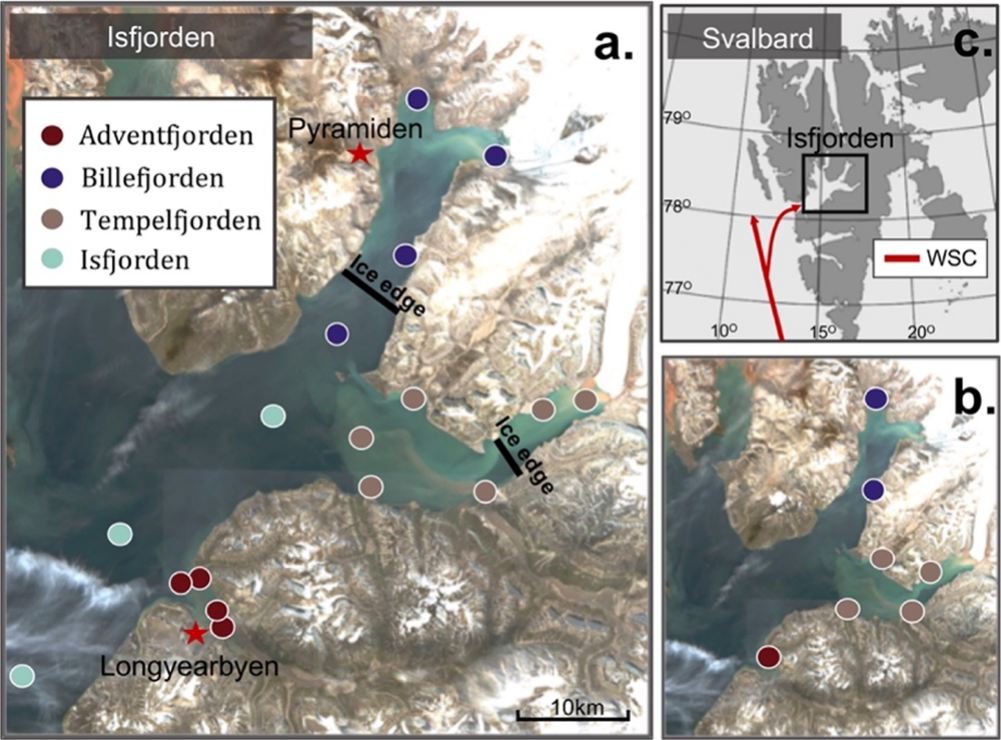
(a) Satellite image (Copernicus Sentinel data [August 20, 2018]) of Isfjorden where zooplankton were sampled in May, June, and August 2018 and benthic invertebrates in August 2018. The position of the ice edge in May 2018, when land-fast ice prevented sampling at the innermost stations, is indicated in black. Stars represent the city of Longyearbyen and the abandoned mining village of Pyramiden, which represent local sources of contamination. (b) Isfjorden station map showing stations where sculpin were sampled using gillnets in August 2018. (c) Map of Svalbard with the West Spitsbergen Current (WSC) depicted in red.

Zooplankton, benthic invertebrates, and sculpin, as well as temperature and salinity profiles and surface water samples, were collected from 17 stations in Isfjorden (Adventfjorden, Tempelfjorden, and Billefjorden) in 2018 (Figure 1). Zooplanktons were sampled spatially and seasonally in May (10–11), June (18–24), and August (16–24), while benthic invertebrates and sculpin were sampled spatially in late summer (August 24–September 1). Fjord stations were positioned along gradients from river estuaries and glacier fronts to the outer fjord (Figure 1). Glacier front stations in Billefjorden and Tempelfjorden were inaccessible in May due to the presence of land-fast ice. Methods for collection and analysis of environmental data, including water mass determination, salinity, temperature, and turbidity, are described, along with results in a parallel study.^22^

A range of vertical plankton net (WP) sizes were used for zooplankton collection, including WP2 (0.25 m^2^ diameter with 60 and 200 μm mesh sizes) and a larger and coarser WP3 (1 m^2^ diameter with 1000 μm mesh size). Net contents were pooled and macrozooplankton were selectively removed and frozen separately. The rest of the pooled zooplankton were size-fractionated through 500 and 1000 μm sequential Nitex mesh screens.

Benthic invertebrates were sampled using a Van Veen grab from the same fjord stations as the zooplankton (Figure 1a), while sculpin were sampled from river estuaries and other nearshore stations using gill nets deployed at 10–15 m depth (Figure 1b). Samples were homogenized, and subsamples of macro- and size-fractionated zooplankton, benthic invertebrates (whole organisms), and sculpin (dorsolateral muscle tissue) were frozen (−20 °C) separately for contaminant [in solvent-rinsed, precombusted (450 °C, 6 h) glass containers] and stable isotope (δ^13^C and δ^15^N) analyses. In addition, subsamples of zooplankton size fractions were fixed (4% buffered formaldehyde–seawater solution) for species identification and abundance-based compositional determination (Figure S1).

### 2.3 Stable Isotope Analysis

Bulk stable isotope analysis of carbon and nitrogen (δ^13^C and δ^15^N) was carried out on zooplankton (*n* = 44) and benthic invertebrates (*n* = 24) at the University of California, Davis (UC Davis Stable Isotope Facility, USA), while sculpin (*n* = 27) samples were analyzed at the University of Oslo (UiO Stable Isotope Laboratory). All samples were freeze-dried, homogenized, weighed, and packed in tin capsules prior to analysis. Samples were not lipid-extracted. Subsamples of benthic organisms expected to have a high calcium carbonate content (mollusks and echinoderms) were acidified to remove inorganic carbon. Due to potential impacts of acidification on δ^15^N values,^34^ acidified samples (used for δ^13^C values) were analyzed in parallel with unacidified samples (used for δ^15^N values). δ^13^C and δ^15^N were measured using an elemental analyzer interfaced to an isotope ratio mass spectrometer.^35^ Long-term standard deviations at UC Davis are 0.2‰ for δ^13^C and 0.3‰ for δ^15^N. Run-specific standard deviations at UiO were 0.04‰ for δ^13^C and 0.02‰ for δ^15^N. Stable carbon and nitrogen isotope values are expressed using delta notation, relative to international standards (Vienna PeeDee Belemnite for C, and atmospheric N for nitrogen).^36^

### 2.4 Contaminant Analysis

Contaminant analyses were carried out at the Norwegian Institute for Air Research’s (NILU) laboratory in Tromsø, Norway. Zooplankton (*n* = 44), benthic invertebrates (*n* = 26), and sculpin (*n* = 35) were analyzed for HCB and PCBs (CB-28, 31, 52, 101, 118, 138, 153, and 180). In addition, all zooplankton (*n* = 44) and several benthic invertebrates (*n* = 10) were analyzed for DDTs (*o*,*p*′- and *p*,*p*′-DDT) and their metabolites (*o*,*p*′, *p*,*p*′-DDE and -DDD), as well as α-, β-, γ-HCH, cis- and trans isomers for chlordane and nonachlor, and mirex. CB-28 and 31 coeluted and are treated together. In addition, all zooplankton samples were further analyzed for EFs [EF = +/(+&−)] of chiral α-HCH, *trans*- and *cis*-chlordane.

All equipment was precombusted and solvent-washed. All chemicals were SupraSolv grade (Merck). Zooplankton, benthic invertebrates, and sculpin samples were extracted and analyzed according to previously described methods.^37^ Briefly, samples were homogenized, weighed, and freeze-dried in 1:3 (w/w) Na_2_SO_4_ (precombusted at 600 °C) overnight. The following day, ^13^C-labeled internal standards (HCB, PCB-28, PCB-31, PCB-52, PCB-101, PCB-118, PCB-138, PCB-153, PCB-180, α-HCH, β-HCH, γ-HCH, *p*,*p*′-DDE, *p*,*p*′-DDD, *p*,*p*′-DDT, *trans*-chlordane, *cis*-chlordane, *trans*-nonachlor) were added to the samples before 15 min of ultrasonic extraction with 3:1 (v/v) cyclohexane/acetone. The solvent phase was isolated and evaporated in preweighed vials for gravimetric lipid determination. Lipids were then removed using solid phase extraction (EZ-POP columns (Supelco/Merck) eluted with acetonitrile) and additional cleanup using precombusted florisil (450 °C). Samples were then evaporated and transferred to a GC vial, and the recovery standard (^13^C-labeled CB-159) was added. Target analytes were analyzed using gas chromatography high-resolution accurate mass spectrometry (GC-HRAM) using a GC-Q-Exactive Orbitrap mass analyzer (Thermo Scientific, UK). Cold splitless injection using programmable temperature vaporization (PTV) with a 1 μL injection volume was performed. The PTV injector was held at 90 °C for 0.15 min, ramped to 320 °C at 5 °C/min with a hold time of 5 min. Details surrounding chromatographic separation and mass spectrometer settings are previously described by Warner and Cojocariu.^38^

Quality assurance of the analytical method was assessed through measurements of laboratory blanks (15 procedural blanks) and standard reference material (contaminated fish; EDF-2524, Cambridge Isotope Laboratories, UK). Samples were blank-corrected. The limit of detection (LOD) and quantification (LOQ) were defined as 3 and 10 times the standard deviation of the blank replicates for each extraction batch, respectively. The LOD ranged from 0.01 to 47.0 pg g^–1^ ww for the POPs analyzed (Table S1), and average recovery for the ^13^C-labeled compounds ranged from 9.6 to 110.1% for biota samples and from 11.9 to 68.3% for standard reference material (Table S2).

Enantiomer selective analysis of α-HCH and *cis*- and *trans*-chlordane in zooplankton samples was performed using a chiralsil-dex column [12.5 m × 0.25 mm × 0.25 μm (Agilent (chrompack), USA)] connected in tandem with a TG5-SILMS [12.5 m × 0.25 mm × 0.25 μm (Thermo Scientific, UK)]. Analysis was performed on a TSQ 9000 GC–MS/MS (Thermo Scientific, UK) using a 2 μL injection volume with conditions described previously using PTV injection. Ion transitions with collision energies, chromatograph separation, and mass spectrometer conditions are described in Table S3 of the Supporting Information. The baseline racemic range was defined as the average EF ± the standard deviation of the standards; α-HCH (0.51–0.51), *trans*-chlordane (0.51–0.52), and *cis*-chlordane (0.49–0.50).

### 2.5 Data Analyses

Statistical analyses were performed using R version 4.0.2 (R Development Core Team, 2020). Individual compounds that were detected in less than 60% of the samples (CB-118, CB-138, CB-180, *o*,*p*′-DDT and mirex for zooplankton, CB-28/31, CB-101 and CB-118 for sculpin and γ-HCH, *o*,*p*′-DDT, and *p*,*p*′-DDD for benthic invertebrates) were removed from the analysis. For the remaining congeners, nondetects were replaced with values (assuming a beta distribution; α = 5, β = 1) conditioned to fall between 0 and LOD using a multiple imputation method.^39^ Replaced values represent 12% (for PCBs/HCB) and 8% (for other analyzed pesticides) of the zooplankton values, 24% (for PCBs/HCB) and 19% (other pesticides) of the benthic invertebrate values, and 16% (PCBs/HCB) of the sculpin values. In addition, all contaminant groups are summed and presented as ∑POPs in order to visualize main trends in contaminant loads of coastal fauna.

To investigate the relationships between POP concentrations and stable isotopes, lipids, sampling date, taxonomic grouping, and sampling location, Wilcoxon rank sum tests or Kruskal–Wallis rank sum test with the post hoc Dunn’s test^40^ were performed to account for non-normal distributions (*p* < 0.05, Shapiro–Wilk’s test).^41^*P*-values were adjusted for multiple comparisons using the Bonferroni correction.^42^ In consideration of our small sample sizes and skewed data, results are presented as bootstrapped means with 95% confidence intervals.^43^ Seasonality in zooplankton contaminant loads occur alongside seasonal changes in lipid content, so results are given in ng/g lipid weight (lw) for zooplankton. Sculpin and benthic invertebrates, however, were only sampled spatially. Thus, due to unusually low gravimetrically determined lipid weights from Adventfjorden sculpin, results for both sculpin and benthic invertebrates are provided on a wet weight (ww) basis for better comparison among fjords.

Water chemistry data collected from two depths (surface and 15 m)^22^ were averaged for each station to be used in relation to zooplankton collected from the entire water column. To account for seasonal variation in lipid content (range: 0.2–6.4%), zooplankton δ^13^C values were lipid-corrected based on their CN ratios (range: 2.2–7.6), using the model proposed by Pomerleau et al. (2014).^44^ Sculpin and benthic invertebrates had a low lipid content (<3%), so δ^13^C values were not lipid-corrected for these groups.^45^

Redundancy analysis (RDA) was carried out in the R package “vegan”^46^ to evaluate the importance of physical and ecological drivers for explaining variance in contaminant concentrations in zooplankton, sculpin, and benthic invertebrates separately. Prior to RDA analyses, contaminant mass fractions were log-transformed to reduce skewness and the influence of abundant congeners on the outcome of the ordination. For herbivorous zooplankton, partial RDA was carried out on the sums of contaminant groups with lipid content included as a covariable. Scaled explanatory variables were grouped according to four likely seasonal drivers of contaminant accumulation: (1) terrestrial inputs were represented by salinity, (2) carbon source by zooplankton δ^13^C, (3) seasonal atmospheric volatilization by surface water temperature. To check for multicollinearity among explanatory variables, variance inflation factors were calculated to confirm that VIFs were <5.^47^ Variance partitioning was then carried out using a series of partial RDAs, in order to better understand the degree of overlapping variance among the four drivers (terrestrial inputs, carbon source, temperature, and changes in lipid content).

For benthic invertebrates, partial RDA was carried out using lipid content (which was significant for explaining variance in the POP content of zoobenthos) as a covariable. Explanatory variables included δ^13^C and δ^15^N, feeding habit, taxonomic group, fjord, and sampling location (to represent distance to rivers/glaciers). To test the impact of local contaminant loads on invertebrate contaminant concentrations, sediment ∑_8_PCB and HCB concentrations (using published data from the same fjords; from Johansen et al.)^23^ were included as explanatory variables. For sculpin, partial RDA was carried out with fish length included as a covariable. Both fjord and location (estuary vs nearshore) were included as environmental variables, δ^13^C and δ^15^N as food web tracers and sediment ∑_8_PCB and HCB content as indicators of local contamination. With variance explained by covariables removed, partial RDA models fit the leftover explanatory variables to the residual variance. To test the significance of these models, permutation tests (Monte-Carlo, 10,000 permutations; significance level of *p* ≤ 0.05) were run on the model residuals.

## 3 Results

### 3.1 Characteristics of Sampled Fauna

Zooplankton collected for POP analysis included both size-fractionated samples (“size fractions”) and individual taxa. Zooplankton size fractions were dominated by herbivorous zooplankton. In May, size fractions were dominated by *Cirripedia nauplii* and decapoda larvae (zoea), while copepodites of *Calanus* spp. were prevalent in June and August (Figure S1). Individual macrozooplankton taxa consisted of predator chaetognaths (*Parasagitta elegans* and *Eukrohnia hamata*), the small fish *Leptoclinus maculatus,* as well as the omnivorous euphausiid *Thysanoessa* spp in May and June. In August, predator jellyplankton, including *Mertensia ovum*, *Beroe cucumis,* and *Cyanea capillata*, were also present (Table S5).

The lipid content in herbivorous zooplankton increased from May (1.63, CI: 1.21–2.07% ww) to August (3.19, CI: 2.11–4.15% ww; Dunn’s: *p* = 0.05), while lipids in omnivorous/predator zooplankton remained similar between these months (Wilcoxon: *p* = 0.121). Lipid-corrected δ^13^C values decreased seasonally in herbivorous zooplankton, indicating a shift from marine to terrestrial carbon from May (−19.68, CI: −20.45 to −18.98‰) to June (−21.77, CI: −22.44 to −21.2‰; Dunn’s: *p* = 0.005) and to August (−24.31, CI: −24.71 to −23.84‰; Dunn’s: *p* = 0.005; Figure S2 and Table 1).^36^ Values of δ^15^N were higher in omnivorous/predator zooplankton (9.8, CI: 8.72–11.03‰) than in herbivorous zooplankton (7.73, CI: 7.45–8.03‰; Wilcoxon: *p* = 0.001) but did not differ among months within each feeding group (Kruskal–Wallis: *p* > 0.05, Figure S2).

**Table 1:** Summary Statistics of Sample Means and 95% CI for Zooplankton^a^

			lipid	δ^13^C	∑_8_PCB	HCB	∑DDT	∑chlordanes	∑HCH	
feeding group	month	*n*	(%)	(‰)	(ng g^–1^ lw)	(ng g^–1^ lw)	(ng g^–1^ lw)	(ng g^–1^ lw)	(ng g^–1^ lw)	EF-αHCH
herbivores	May	8	1.63 (1.21–2.04)	–19.68 (−20.38 to −18.94)	4.43 (2.75–6.31)	14.9 (10.45–18.87)	4.77 (3.16–6.73)	3.54 (2.18–5.16)	1.3 (0.88–1.7)	0.39 (0.38–0.39)
	June	16	1.58 (1.25–1.98)	–21.77 (−22.48 to −21.18)	2.52 (2.07–3.01)	4.47 (3.86–5.1)	2.6 (2.17–3.11)	1.98 (1.74–2.24)	1.18 (1.06–1.29)	0.41 (0.39–0.42)
	August	8	3.19 (2.2–4.13)	–24.31 (−24.71 to −23.82)	1.6 (1.3–1.93)	1.62 (1.42–1.88)	2.1 (1.75–2.48)	1.54 (1.42–1.63)	1.57 (1.45–1.72)	0.41 (0.4–0.43)
omnivores/predators	May	3	1.91 (0.67–3.72)	–21.29 (−22.45 to −20.08)	6.91 (5.04–9.78)	21.38 (15.76–31.7)	9.46 (6.27–14.72)	11.1 (8.69–15.85)	1.25 (0.56–1.74)	0.39 (0.39–0.39)
	August	11	1.36 (0.63–2.53)	–21.86 (−22.57 to −21.18)	4.8 (2.12–9.16)	6.72 (3.81–10.69)	2.61 (1.55–3.87)	2.7 (1.69–4.01)	1.08 (0.73–1.44)	0.41 (0.39–0.44)

a Zooplankton samples collected by fjord (and month) included *n* = 6 in Adventfjorden (May: 2, June: 4, Aug: 0), *n* = 8 in Billefjorden (May: 1, June: 3, Aug: 4), *n* = 14 in Tempelfjorden (May: 3, June: 5, Aug: 6), and *n* = 18 in outer Isfjorden (May: 5, June: 4, Aug: 9).

Sampled benthic taxa included filter/suspension feeders (the bivalve *Astarte* spp., *Cilliatocardium cilliatum*, *Serripes groenlandicus*, *Mya arenaria,* and *ascidians*), surface-deposit and deep-deposit feeders (bivalve *Macoma calcarea* and polychate *Maldane sarsi,* respectively), predators (polychaete *Nephtys* sp. and decapods *Pandalus borealis* and *Sabinea septemcarinata*), and scavengers (seastar *Leptasterias muelleri* and crab *Hyas araneus*). Due to a lack of adequate replication at the species level, benthic invertebrates were grouped by these feeding strategies for comparison among and within fjords (Table S6). Lipid content (0.9; CI: 0.64–1.17%) and δ^13^C values (−20.53; CI: −21.07 to −20.05‰) in benthic invertebrates did not differ among fjords or feeding groups (Kruskal–Wallis: *p* > 0.05) except for those in Adventfjorden, where sampled ascidians had a relatively low lipid content. Values of δ^15^N were higher in predator species (11.09, CI: 10.58–11.61‰) compared to filter feeders and surface-deposit feeders (8.18, CI: 7.27–9.13‰; Wilcoxon: *p* < 0.001; Figure S3).

For shorthorn sculpin (*Myoxocephalus scorpius*), individuals collected from gillnets were mostly female (32 female, 3 male) with a mean length of 19.9 cm (CI: 19.1–20.7) and mean weight of 165 g (CI: 142.5–188.3). The sculpin lipid content was lower in Adventfjorden (0.02, CI: 0.01–0.02%) than in Billefjorden (0.5, CI: 0.2–0.9%) and Tempelfjorden (0.4, CI: 0.1–0.8%). Values of δ^13^C (−19.24, CI: −19.5 to −19.01‰) did not differ among fjords (Kruskal–Wallis: *p* > 0.05). Values of δ^15^N were higher in Billefjorden (14.27, CI: 14–14.61‰) compared to those in Adventfjorden (13.39, CI: 13.11–13.59‰; Dunn’s: *p* = 0.048) and Tempelfjorden (13.41, CI: 13.03–13.81‰; Dunn’s: *p* = 0.01; Figure S4).

### 3.2 POP Concentrations in Isfjorden Biota

HCB concentrations (on a wet weight basis) in zooplankton ranged from 0.03 to 0.59 ng/g ww (May: 0.27, CI: 0.18–0.35 ng/g ww, June: 0.06, CI: 0.05–0.07 ng/g ww, and August: 0.07, CI: 0.04–0.12 ng/g ww). After lipid normalization, HCB concentrations ranged from 1.28 to 31.70 ng/g lw (May: 16.67, CI: 12.44–20.93 ng/g lw; June: 4.47, CI: 3.84–5.07 ng/g lw; and August: 4.57, CI: 2.61–7.36 ng/g lw). ∑_8_PCB concentrations (on a wet weight basis) in zooplankton ranged from 0.01 to 0.19 ng/g ww (May: 0.08, CI: 0.05–0.11 ng/g ww, June: 0.04, CI: 0.03–0.05 ng/g ww, and August: 0.05, CI: 0.03–0.07 ng/g ww). After lipid normalization, ∑_8_PCB concentrations ranged from 0.96 to 26.06 ng/g lw (May: 5.11, CI: 3.62–6.80 ng/g lw; June: 2.52, CI: 2.09–2.99 ng/g lw; and August: 3.45, CI: 1.82–6.23 ng/g lw).

**Figure 2:**
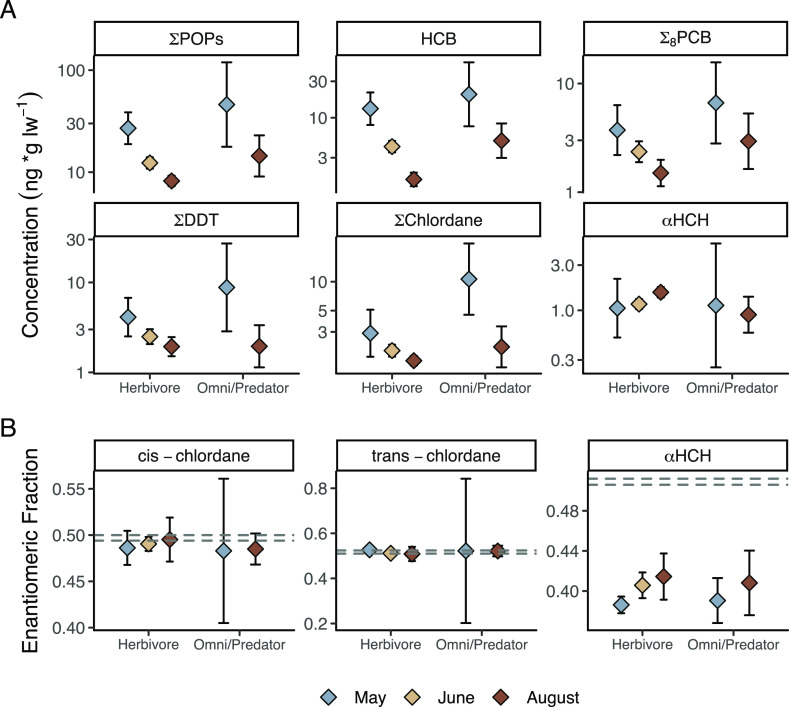
(A) POP concentrations and (B) EFs in bulk zooplankton by month for each plankton type: herbivorous zooplankton (*Calanus* spp., Meroplankton) and omnivorous and predator zooplankton (Macrozooplankton and Jellyplankton). Diamonds and error bars represent the bootstrapped mean and 95% confidence interval. ∑_8_PCB is defined as the sum of CB-28, CB-31, CB-52, CB-101, and CB-153 (CB-118, CB-138, and CB-180 were <LOD in zooplankton). The racemic ranges (determined using laboratory standards) are indicated as dashed gray lines. POP concentrations on a wet weight basis can be found in Figure S8.

To facilitate interpretation, data were pooled by the feeding group for further statistical analysis and visualization (*Calanus* spp.-, *Cirripedia nauplii*-, and decapod zoea-dominated size fractions as herbivores and individual macrozooplankton and jellyplankton as omnivores/predators). Contaminant concentrations did not differ among taxa within each feeding group by month (Kruskal–Wallis: *p* > 0.05). In addition, no spatial trends were observed in contaminant concentrations by the feeding group within each month (Kruskal–Wallis tests among fjords within each month: *p* > 0.05; Figure S5). While herbivorous and predatory zooplankton both exhibited similar seasonal trends for each POP group, concentrations were consistently higher in predatory zooplankton (Figure 2a; Wilcoxon rank sum tests for each contaminant group: *p* < 0.05).

Lipid-adjusted ∑POPs in zooplankton decreased from May to August for most contaminant groups (Figure 2a). HCB was the dominant contaminant and demonstrated a seasonal decrease in herbivorous zooplankton from May (14.9, CI: 10.24–18.9 ng/g lw) to June (4.47, CI: 3.87–5.09 ng/g lw) to August (1.62, CI: 1.4–1.89 ng/g lw; Dunn’s: *p* < 0.001; Figure 2a). Similar downward trends were visible for ∑_8_PCB, ∑DDTs, and ∑chlordane pesticides from May to August for both herbivorous and omni/predator zooplankton (Figures 2a and S6; Table 1). This decrease from May to June/August was also apparent on a wet weight basis for both feeding groups (Figures S7 and S8). In contrast, α-HCH concentrations increased from June (1.18, CI: 1.06–1.29 ng/g lw) to August (1.57, CI: 1.44–1.72 ng/g lw) in herbivorous zooplankton (Wilcoxon: *p* = 0.004; Figure 2a and Table 1). An increase from May/June to August was also observed on a wet weight basis for herbivorous zooplankton. In addition, EFs of α-HCH were significantly closer to the racemic range in August (0.41, CI: 0.4–0.43) compared to May (0.39, CI: 0.38–0.39; Wilcoxon: *p* = 0.02; Figure 2b).

∑POPs were higher in scavenger and predator benthic invertebrates compared to filter and deposit feeders (Wilcoxon: *p* = 0.002), especially for the higher chlorinated PCBs (Figure S9). For surface deposit-feeding and filter-feeding zoobenthos, ∑_8_PCB was higher at the outer Isfjorden stations (0.25, CI: 0.16–0.37 ng/g ww) compared to the inner fjord arms (Billefjorden: 0.1, CI: 0.04–0.2 ng/g ww, Adventfjorden: 0.13, CI: 0.04–0.3 ng/g ww, and Tempelfjorden: 0.06, CI: 0.04–0.09 ng/g ww; Table 2). ∑_8_PCB and HCB were highest in sculpin collected from Billefjorden (∑_8_PCB: 0.22, CI: 0.14–0.33 ng/g ww; HCB: 0.1, CI: 0.08–0.12 ng/g ww), with concentrations significantly higher than those from Tempelfjorden (∑_8_PCB: 0.09, CI: 0.06–0.13 ng/g ww; HCB: 0.06, CI: 0.05–0.08 ng/g ww; Wilcoxon: *p* < 0.25; Figure 4 and Table 2).

**Table 2:** Summary Statistics of Sample Means and 95% CI for Benthic Invertebrates (Filter/Deposit Feeders) and Sculpin

			lipid	δ^13^C	δ^15^N	∑_8_PCB	HCB
zoobenthos	fjord	*n*	(%)	(‰)	(‰)	(ng g^–1^ ww)	(ng g^–1^ ww)
filter/deposit feeders	Billefjorden	3	0.54 (0.28–0.7)	–21.8 (−23.12 to −20.82)	6.96 (6.59–7.28)	0.1 (0.04–0.2)	0.08 (0.03–0.16)
	Adventfjorden	3	0.09 (0.02–0.15)	–21.17 (−22.57 to −20.15)	8.53 (6.67–10.24)	0.13 (0.04–0.3)	0.04 (0.04–0.05)
	Tempelfjorden	3	0.60 (0.29–1.07)	–20.9 (−21.7 to −20.36)	7.6 (6.16–10.19)	0.06 (0.04–0.09)	0.05 (0.02–0.09)
	Isfjorden	3	0.36 (0.30–0.44)	–20.23 (−20.5 to −19.95)	9.62 (7.55–10.74)	0.25 (0.16–0.37)	0.15 (0.11–0.19)
sculpin	Billefjorden	9	0.50 (0.20–0.90)	–19.35 (−19.77–18.95)	14.27 (14–14.57)	0.22 (0.13–0.33)	0.1 (0.08–0.12)
	Adventfjorden	3	0.02 (0.01–0.02)	–19.39 (−19.47–19.3)	13.39 (13.11–13.59)	0.08 (0.06–0.1)	0.06 (0.05–0.07)
	Tempelfjorden	18	0.4 (0.10–0.80)	–19.15 (−19.52–18.76)	13.41 (13.03–13.76)	0.09 (0.06–0.13)	0.06 (0.05–0.08)

### 3.3 Physical and Ecological Drivers of Contaminant Concentrations

Seasonality in the physical–chemical environment in Isfjorden is reported in a parallel study (Figure S10).^22^ Briefly, land-fast sea ice was present in Billefjorden and Tempelfjorden in May, and many stations were dominated by local and winter-cooled water (temperature < 1; salinity < 35; Figure S11). High concentrations of chlorophyll-*a* in the water column, coinciding with low nutrient concentrations, suggest that May sampling took place approximately 1 week after the peak of the spring phytoplankton bloom.^22,48^ In June, freshwater from river runoff and glacier-front ablation was detected in surface waters throughout Isfjorden. In August, freshwater inputs to surface waters, alongside Atlantic Water (Figure S11) advection from the West Spitsbergen Current (WSC; Figure 1a,c), resulted in stratification of the water column. In Isfjorden, marine- and land-terminating glaciers deliver freshwater to the fjord, transporting highly suspended sediment loads, terrestrial organic matter, and inorganic nutrients to the fjord.^22^

**Figure 3:**
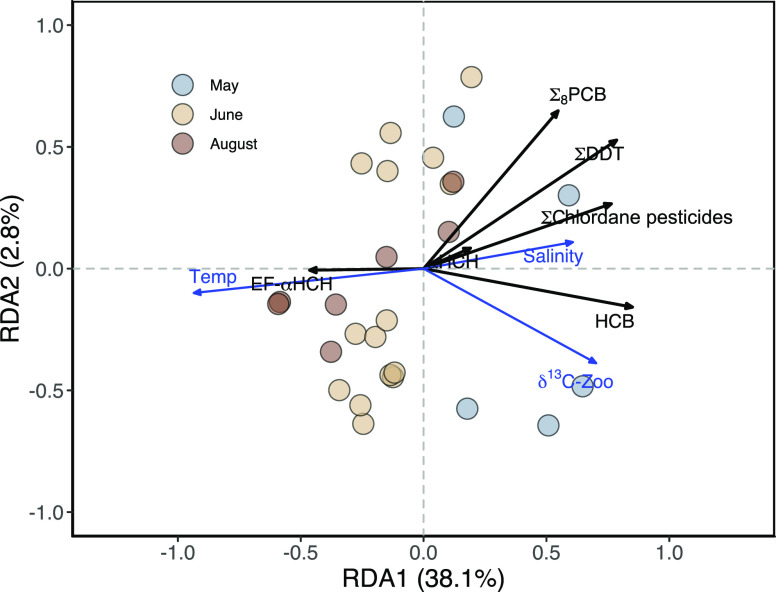
Partial RDA based on log-transformed concentrations of sums of PCBs, chlordane pesticides, DDTs, and α-HCH in herbivorous zooplankton with variance (20.6%) due to lipid content removal. Constraining variables: δ^13^C-Zoo, salinity, and temperature, which explain 41% of the residual variance, are shown in blue. EF of α-HCH (in black) is included as a passive vector. Each point represents one individual sample, and color represents the sampling month with blue = May, light brown = June, and dark brown = August.

In the zooplankton RDA, constraining variables explained a significant amount of the residual variance in herbivorous zooplankton contaminant concentrations (41.0%, permutation test: *p* = 0.001; Figure 3) when variance due to lipid content (20.6%) was removed. The first axis, which separates May from June and August and represents overlapping seasonal and freshwater gradients, explained 38.1% of the variance (permutation test: *p* = 0.001). The second axis, which captures the within-season spatial variability, explained only 2.8% of the variance in zooplankton contaminant concentrations and was not significant (permutation test: *p* > 0.05; Figure 3). Results of variance partitioning illustrate the extensive overlapping variance of the explanatory variables (Figure S12). For benthic invertebrates, the lipid content explained 30.7% of the variance in contaminant concentrations (permutation test: *p* = 0.001). When variance due to lipid content was accounted for, only taxonomic grouping was significant, explaining 55% of the residual variance. For sculpin, fjord and fjord sediment concentrations of ∑_8_PCB were the best predictors of contaminant concentrations, explaining 15.5 and 13.8% of the residual variance, respectively, when variance due to fish length (6.7%) was removed. Other variables, including sampling location in the fjord, and δ^13^C and δ^15^N values were not significant.

## 4 Discussion

### 4.1 Terrestrial Inputs are Associated with Lower Concentrations of ∑POPs in Isfjorden Biota

Climate change-driven increases in temperature are leading to enhanced glacial melt. Here, we investigated the role of glacial meltwater as a secondary source of POPs to coastal food webs along spatial and seasonal gradients in the glacial influence. In Isfjorden, extreme seasonal variations in day length drive seasonal changes on land, where the melt season progresses from snow melt in May and June to glacier melt and permafrost thaw in July and August.^49,50^ This seasonal progression is associated with the delivery of increasingly warm and sediment-laden meltwater to coastal waters either directly through glacier-front ablation or through riverine inputs.^22^

In our study, decreasing water column salinity, increased turbidity, and zooplankton terrestrial carbon utilization were associated with reduced contaminant concentrations, contradicting our hypothesis that glacier meltwater inputs are an important secondary source of legacy POPs to Isfjorden biota. These findings stand in contrast to previous studies on Svalbard, which have attributed increased POP exposure in sediment compartments to meltwater inputs.^51−53^ However, our observations are in agreement with recent findings from Isfjorden, which found that high sediment loads from marine-terminating glaciers and rivers may act to scavenge and/or dilute contaminant concentrations in coastal waters and sediments.^23^

### 4.2 Glacial Meltwater may be a Source of α-HCH to Coastal Zooplankton

While we observed a general decrease in zooplankton contaminant concentrations through the melt season for most POP groups, this was not the case for HCHs. In fact, contaminant profiles demonstrate a clear transition from HCB dominance in May to HCH dominance in August, with α-HCH representing the most prevalent isomer. HCH has a lower octanol–water partitioning coefficient (*K*_ow_) and therefore higher solubility in water compared to the higher *K*_ow_ HCB and PCBs, which are more likely to be bound to inorganic sediments and therefore not as bioavailable for zooplankton in glacial meltwaters.

Enantioselective analysis of α-HCH illustrates the potential role of glaciers as a secondary source of α-HCH to the fjord in late summer. EF signatures in zooplankton were more racemic in August, when the fjord was most impacted by glacial melt, especially at the glacier fronts and river estuary stations.^22^ Historically deposited α-HCH stored in glaciers are not subject to substantial microbial degradation. Thus, in theory, fresh inputs should reflect an EF closer to that of the racemic (equal amounts of left- and right-handed enantiomers) industrial product, while biologically degraded compounds deviate from a racemic signature.^54^ While macrozooplankton degrades chiral POPs enantiomer selectively,^55^ EFs in lower trophic level zooplankton, including *Calanus* spp. and meroplankton, should reflect the chiral signature of the surrounding environment.^31,56^

Thus, the change in α-HCH EFs in zooplankton toward a more racemic signature in August indicates fresh inputs of α-HCH to the fjord from glacial meltwater. Atlantic water advection in August may also be a source of racemic oceanic α-HCH to zooplankton.^31^ However, considering the spatial gradient investigated within this study, EFs were closer to racemic in estuarine zooplankton compared to the outer fjord, and the correlations with salinity and turbidity suggest that freshwater inputs from melting glaciers are likely the main driver of the observed patterns. While atmospheric concentrations of HCH have declined since 1990 in Svalbard and the Canadian Arctic,^57,58^ our results suggest that exposure trends to coastal fauna may be spatially dependent and deviate from atmospheric trends with continued glacial meltwater release of HCHs into Arctic coastal waters.

### 4.3 Physical and Biological Processes Explain Seasonal Decrease in Zooplankton Contaminant Concentrations

POP concentrations in zooplankton were similar or lower compared to previous studies in Svalbard,^59^ the Canadian Arctic,^60,61^ and the marginal sea-ice zone.^32^ Total contaminant concentrations (∑POPs) decreased seasonally in all taxa. However, concentrations in omnivorous/predatory zooplankton were consistently higher compared to herbivorous zooplankton, indicating biomagnification of POPs through the zooplankton food web, as previously described for Arctic zooplankton.^59,62−64^

While glacial inputs were likely a source of α-HCH, all other contaminant groups demonstrated clear and significant seasonal decreases. This seasonal decrease is likely due to seasonality in several processes acting in concert that affect primary production and lipid content in zooplankton, which in turn influence the seasonal availability and uptake of POPs in the food web.^20,24,59,63^ The highest concentrations of POPs in zooplankton were observed in May, during ice breakup, alongside higher δ^13^C values, indicating reliance on marine carbon from the spring phytoplankton bloom.^36^ These findings are in line with previously documented seasonal processes in the Arctic.^65^ During the Arctic polar night, cold temperatures and sea ice can act chemically and physically to prevent outgassing of POPs from the water column, resulting in increased dissolved concentrations.^20^ This is particularly true for highly volatile compounds, like HCB, which has had relatively stable concentrations in the Svalbard atmosphere since 1990^58^ and which dominated zooplankton contaminant profiles in May. Subsequently, with the return of the sun in spring, ice-algae and pelagic phytoplankton blooms commence as surficial snow melts and the sea ice is broken up.^66^ This rapid increase in biomass in the water column provides increased surface area for POPs to adsorb to, a process driven by their high affinity for organic matter.^67,68^ Thus, zooplankton grazing on the spring phytoplankton bloom in May is exposed to higher concentrations of POPs within the water column, as well as through their diet. Similar findings have been reported for littoral amphipods in Adventfjorden.^69^

The decrease in POP concentrations from May to June was observed on both a lipid weight and wet weight basis, suggesting reduced exposure following ice melt and the spring phytoplankton bloom. In contrast, the decrease in contaminant concentrations from June to August on a lipid weight basis was not observed on a wet weight basis. For herbivorous zooplankton, May and June communities were dominated by meroplankton and the lipid-depleted overwintering population of *Calanus* spp. The seasonal increase in relative abundance of *Calanus* spp. in August size fractions, together with accumulation of storage lipids through the summer feeding season, suggests that lower contaminant concentrations from June to August can be attributed to changes in species composition and lipid dilution.^70,71^

### 4.4 Zoobenthos Reflect Impacts of Local Sources and Inorganic Sedimentation

Zoobenthos, including the higher trophic level sculpin, provide a time-integrated perspective on contamination on annual and multiyear time scales. Thus, stationary infauna as well as sculpin, known to be a territorial fish with a small home range,^72^ should reflect the signal in the location collected. While benthic invertebrates and sculpin showed similar concentrations of POPs to previous studies for Svalbard zoobenthos,^69,73,74^ the spatial patterns across the Isfjorden system highlight the importance of inputs from local point sources and effects of fjord-specific physical processes, like varying sedimentation rates, on exposure to the benthic environment.

**Figure 4:**
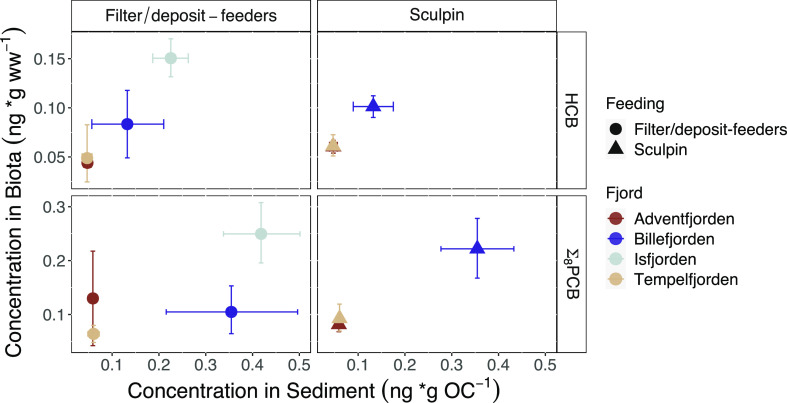
HCB and ∑_8_PCB concentrations in filter- and deposit-feeding benthic invertebrates and sculpin vs fjord sediment concentrations.^23^ Points and error bars represent the bootstrapped mean and 95% confidence intervals based on all fjord replicates. ∑_8_PCB is defined as the sum of CB28/31, CB-52, CB-101, CB-118, CB-138, CB-153, and CB-180 for zoobenthos and CB-52, CB-138, CB-153, and CB-180 for sculpin.

The sampling design employed here targeted the contrast between river estuaries and marine-influenced areas of the fjord with the aim of distinguishing impacts of river runoff and associated shifts in the carbon source on contaminant loads. However, no difference between within-fjord sampling locations was detected, and spatial differences in δ^13^C values in biota had no effect on PCB or HCB concentrations. Instead, the sampled fjord was the most important explanation for HCB and PCB contamination in sculpin and lower trophic level benthic invertebrates (filter and surface deposit feeders). The high POP concentrations in Billefjorden fauna reflect the impact of the previously described point source from the Russian mining settlement Pyramiden, which was closed in 1997.^23,75−78^ POP concentrations in Billefjorden sediments sampled adjacent to Pyramiden are fivefold higher than Adventfjorden and Tempelfjorden sediments.^23^ In contrast, Adventfjorden and Tempelfjorden do not contain significant local sources of PCBs, and lower concentrations match the lower contaminant load in sediment samples collected from the same stations (Figure 4).^23,68^ In addition, Tempelfjorden has a marine-terminating glacier, which delivers high inorganic suspended sediment loads to the fjord. In fact, the highest concentrations in benthic invertebrates were measured from outer Isfjorden, suggesting that oceanic transport of legacy POPs is likely more important than sources associated with glacial meltwater. High sedimentation rates accompanying glacial melt likely dilute sediment contaminant concentrations, creating a spatial gradient along the fjord axis, a process supported by previously reported patterns in sediment concentrations.^23^

### 4.5 Future Perspectives

As temperatures increase globally and glacier mass balance is significantly reduced,^79^ there is concern that coastal areas will increasingly receive inputs of remobilized legacy contaminants from melting cryospheric compartments,^5,12,80^ especially in Arctic regions, where contaminants accumulate due to global distillation processes.^81^ While ice profiles from Svalbard glaciers have illustrated the storage of legacy POPs through the decades,^8,9^ our results do not indicate that these glaciers are an important source of legacy contaminants in coastal fauna. For the benthic compartment, glacial inputs of contaminants are diluted by high rates of inorganic sedimentation, which also likely act to bury local contamination. In the water column, we found indications of accumulation of remobilized α-HCH in coastal zooplankton, but the resulting concentrations were low. All other POP groups, including PCBs, chlordane pesticides, and DDTs, were not associated with glacial meltwater and demonstrated a clear seasonal decline in coastal zooplankton following the spring phytoplankton bloom. For these heavily glaciated Svalbard fjords, other physical and ecological processes, including increased inorganic sediment loads and seasonal lipid accumulation in zooplankton, result in lower contaminant loads during the melt season, outweighing any inputs from glacial melt.

## Supporting Information Available

Target chemicals and CAS numbers; summary of detection limits and concentrations; summary of recoveries; chiral analysis description; ion transitions and collision energies; macrozooplankton and zooplankton size fractions and zoobenthic taxa analyzed for contaminant concentrations; relative abundance of main zooplankton taxa; δ13C, δ15N, and lipid content in zooplankton feeding groups; lipid content, wet weight, δ13C, and δ15N of benthic invertebrates; lipid content, fish length, wet weight, δ13C, and δ15N of sculpin; spatial patterns in herbivorous zooplankton contaminant concentrations; compositional contaminant profiles for herbivorous zooplankton; congener- and isomer-specific contaminant concentrations in zooplankton on the lipid-weight basis and wet-weight basis; composition of ΣPCB8 in benthos from each fjord grouped by feeding habit; and environmental variables used in RDA analysis (PDF)

## Notes

Data Availability: Contaminant data are openly available on DataverseNO (UiT Open Research Data).^82^ The supporting environmental data are published by McGovern et al. (2020).^22^
