# Supplementary material for: Is Glacial Meltwater a Secondary Source of Legacy Contaminants to Arctic Coastal Food Webs?
Source: Environ Sci Technol. 2022 Apr 26;56(10):6337–48. doi: 10.1021/acs.est.1c07062 (PMC9118541; doi:10.1021/acs.est.1c07062)
Supplement: Supplementary file 1 — es1c07062_si_001.pdf [file es1c07062_si_001.pdf]

Supporting Information

Is glacial meltwater a secondary source of legacy contaminants to Arctic coastal food-webs?

Maeve McGovern[1,2,3]*, Nicholas A. Warner[4,5,6], Katrine Borgå[7,8], Anita Evenset[2,9], Pernilla Carlsson[1], Emelie Skogsberg[10,11], Janne E. Søreide[3], Anders Ruus[7,11], Guttorm Christensen[9], Amanda E. Poste[1,2]

[1]Norwegian Institute for Water Research, 9007 Tromsø, Norway, [2]Department of Arctic Marine Biology, UiT, The Arctic University of Norway, 9019 Tromsø, Norway, [3]University Centre on Svalbard, 9170 Longyearbyen, Norway, [4]NILU-Norwegian Institute for Air Research, The Fram Centre, 9007 Tromsø, Norway, [5]Department of Chemistry, UiT, The Arctic University of Norway, 9019 Tromsø, Norway, [6] Thermo Fischer Scientific, 28199 Bremen, Germany, [7]Department of Biosciences, University of Oslo, 0316 Oslo, Norway, [8]Centre for Biogeochemistry in the Anthropocene (CBA), University of Oslo, 0316 Oslo, Norway, [9]Akvaplan-niva, Fram Centre, 9007 Tromsø, Norway, [10]Norwegian University of Life Sciences, Faculty of Environmental Sciences and Natural Resource Management, 1430 Ås, Norway, [11]Norwegian Institute for Water Research, 0579 Oslo, Norway

*Corresponding author: maeve.mcgovern@niva.no

Content Summary:

23 pages

6 Tables

12 Figures

Table S1. Table presenting the target chemicals and add CAS numbers.

Compound name	CAS#
α-Hexachlorocyclohexane (HCH)	319-84-6
β-HCH	319-85-7
γ-HCH	58-89-9
trans (γ)-chlordane	5103-74-2
cis (α)-chlordane	5103-71-9
trans-nonachlor	39765-80-5
cis-nonachlor	5103-73-1
Hexachlorobenzene (HCB)	118-74-1
p,p'-dichlorodiphenyltrichloroethane(DDT)	50-29-3
o,p',-DDT	789-02-6
p,p'-dichlorodiphenyldichloroethane(DDD)	72-54-8
o,p',-DDD	53-19-0
p,p'-dichlorodiphenyltrichloroethylene (DDE)	72-55-9
o,p',-DDE	3424-2-6
Mirex	2385-85-5
Polychlorinated biphenyl (PCB) 28	7012-37-5
PCB 31	16606-02-3
PCB 52	35693-99-3
PCB 101	37680-73-2
PCB 118	31508-00-6
PCB 138	35065-28-2
PCB 153	35065-27-1
PCB 180	35065-29-3

Table S2. Summary of detection limits (LOD) and concentrations for each compound and each sample group. Mean concentrations are calculated using imputed values. Compounds which fell below 60% detection and were removed from analysis are indicated in the 'removed' column.

Group	Compound	Det-ected (%)	Re mo ved	n	LOD (ng/g) (range)	LOD (ng/g) (mean ± sd)	Concentration (ng/g ww) Range	Concentration (ng/g ww) (mean ± sd)
Zooplankton	HCB	100		46	0.001 - 0.016	0.005 ± 0.002	0.011 - 0.586	0.114 ± 0.124
Zooplankton	PCB_101	91.3		46	0.001 - 0.013	0.004 ± 0.002	<LOD - 0.054	0.013 ± 0.011
Zooplankton	PCB_118	30.4	Yes	46	0.002 - 0.038	0.011 ± 0.005	<LOD - 0.03	0.016 ± 0.008
Zooplankton	PCB_138	30.4	Yes	46	0.002 - 0.047	0.014 ± 0.006	<LOD - 0.044	0.021 ± 0.01
Zooplankton	PCB_153	65.2		46	0.001 - 0.031	0.009 ± 0.004	<LOD - 0.138	0.028 ± 0.027
Zooplankton	PCB_180	32.6	Yes	46	0 - 0.011	0.003 ± 0.001	<LOD - 0.022	0.007 ± 0.005
Zooplankton	PCB_28_31	91.3		46	0 - 0.005	0.001 ± 0.001	<LOD - 0.02	0.006 ± 0.004
Zooplankton	PCB_52	91.3		46	0 - 0.007	0.002 ± 0.001	<LOD - 0.065	0.014 ± 0.011
Zooplankton	aHCH	100		46	0.0001 - 0.0001	0.0001 ± 0	0.001 - 0.086	0.023 ± 0.021
Zooplankton	bHCH	82.6		46	0.0001 - 0.0001	0.0001 ± 0	<LOD - 0.04	0.011 ± 0.011
Zooplankton	cis-chlordane	100		46	0.0001 - 0.0001	0.0001 ± 0	0.001 - 0.187	0.027 ± 0.033
Zooplankton	cis-nonachlor	97.8		46	0.0001 - 0.0001	0.0001 ± 0	<LOD - 0.044	0.008 ± 0.009
Zooplankton	gHCH	73.9		46	0.0001 - 0.0001	0.0001 ± 0	<LOD - 0.022	0.007 ± 0.005
Zooplankton	mirex	6.5	Yes	46	0.0001 - 0.0001	0.0001 ± 0	<LOD - 0.001	0.001 ± 0.001
Zooplankton	opDDD	89.1		46	0.0001 - 0.0001	0.0001 ± 0	<LOD - 0.045	0.005 ± 0.008
Zooplankton	opDDT	56.5	Yes	46	0.0001 - 0.0001	0.0001 ± 0	<LOD - 0.015	0.004 ± 0.004
Zooplankton	ppDDD	95.7		46	0.0001 - 0.0001	0.0001 ± 0	<LOD - 0.06	0.008 ± 0.011
Zooplankton	ppDDE	84.8		46	0.002 - 0.017	0.006 ± 0.005	<LOD - 0.116	0.032 ± 0.03
Zooplankton	ppDDT	84.8		46	0.0001 - 0.0001	0.0001 ± 0	<LOD - 0.018	0.003 ± 0.004
Zooplankton	trans-chlordane	100		46	0.0001 - 0.0001	0.0001 ± 0	0 - 0.117	0.021 ± 0.022
Zooplankton	trans-nonachlor	100		46	0.0001 - 0.0001	0.0001 ± 0	0.002 - 0.136	0.022 ± 0.026
Benthos	HCB	100		26	0.002 - 0.009	0.005 ± 0.001	0.02 - 0.89	0.197 ± 0.185
Benthos	PCB_101	80.8		26	0.003 - 0.015	0.005 ± 0.002	<LOD - 0.066	0.031 ± 0.02
Benthos	PCB_118	61.5		26	0.008 - 0.02	0.013 ± 0.003	<LOD - 0.267	0.069 ± 0.073

Benthos	PCB_138	61.5		26	0.003 - 0.025	0.015 ± 0.004	<LOD - 0.268	0.059 ± 0.064
Benthos	PCB_153	76.9		26	0 - 0.016	0.01 ± 0.003	<LOD - 0.503	0.097 ± 0.128
Benthos	PCB_180	69.2		26	0.001 - 0.006	0.003 ± 0.001	<LOD - 0.179	0.031 ± 0.044
Benthos	PCB_28_31	76.9		26	0.001 - 0.003	0.002 ± 0	<LOD - 0.027	0.007 ± 0.006
Benthos	PCB_52	80.8		26	0.001 - 0.004	0.002 ± 0.001	<LOD - 0.027	0.011 ± 0.007
Benthos	aHCH	100		10	0.0001 - 0.0001	0.0001 ± 0.0001	0.001 - 0.178	0.05 ± 0.062
Benthos	bHCH	60		10	0.0001 - 0.0001	0.0001 ± 0.0001	<LOD - 0.043	0.018 ± 0.017
Benthos	cis-chlordane	90		10	0.0001 - 0.0001	0.0001 ± 0.0001	<LOD - 0.063	0.02 ± 0.02
Benthos	cis-nonachlor	80		10	0.0001 - 0.0001	0.0001 ± 0.0001	<LOD - 0.053	0.022 ± 0.021
Benthos	gHCH	40	Yes	10	0.0001 - 0.0001	0.0001 ± 0.0001	<LOD - 0.041	0.022 ± 0.018
Benthos	mirex	70	Yes	10	0.0001 - 0.0001	0.0001 ± 0.0001	<LOD - 0.011	0.003 ± 0.004
Benthos	opDDD	60		10	0.0001 - 0.0001	0.0001 ± 0.0001	<LOD - 0.005	0.002 ± 0.002
Benthos	opDDT	10	Yes	10	0.0001 - 0.0001	0.0001 ± 0.0001	<LOD - 0.003	0.003 ± NA
Benthos	ppDDD	40	Yes	10	0.0001 - 0.0001	0.0001 ± 0.0001	<LOD - 0.014	0.007 ± 0.007
Benthos	ppDDE	90		10	0.005 - 0.005	0.005 ± 0	<LOD - 0.405	0.1 ± 0.135
Benthos	ppDDT	60		10	0.0001 - 0.0001	0.0001 ± 0.0001	<LOD - 0.017	0.004 ± 0.006
Benthos	trans-chlordane	100		10	0.0001 - 0.0001	0.0001 ± 0.0001	0.001 - 0.041	0.018 ± 0.015
Benthos	trans-nonachlor	100		10	0.0001 - 0.0001	0.0001 ± 0.0001	0.001 - 0.148	0.056 ± 0.052
Sculpin	HCB	100		30	0.001 - 0.004	0.003 ± 0.001	0.022 - 0.145	0.073 ± 0.036
Sculpin	PCB_101	33.3	Yes	30	0.006 - 0.013	0.009 ± 0.003	<LOD - 0.093	0.052 ± 0.025
Sculpin	PCB_118	33.3	Yes	30	0.014 - 0.022	0.019 ± 0.002	<LOD - 0.257	0.14 ± 0.08
Sculpin	PCB_138	63.3		30	0.002 - 0.02	0.013 ± 0.008	<LOD - 0.192	0.06 ± 0.051
Sculpin	PCB_153	96.7		30	0 - 0.008	0.005 ± 0.003	<LOD - 0.228	0.066 ± 0.057
Sculpin	PCB_180	93.3		30	0.001 - 0.004	0.003 ± 0.002	<LOD - 0.058	0.018 ± 0.015
Sculpin	PCB_28_31	53.3	Yes	30	0.001 - 0.002	0.002 ± 0.001	<LOD - 0.004	0.003 ± 0.001
Sculpin	PCB_52	66.7		30	0 - 0.002	0.001 ± 0.001	<LOD - 0.019	0.008 ± 0.005

Table S3: Summary of recoveries for 13-C labelled internal standards in biota, lab blanks and standard reference materials (SRMs).

Group	Compound	n	Recovery (%) (mean ± SD)	Recovery (%) (range)
Zooplankton	13C_HCB	42	31.12 ± 8.77	10.94 - 45.77
Zooplankton	13C_PCB101	42	50.38 ± 14.17	15.77 - 79.14
Zooplankton	13C_PCB118	42	51.52 ± 13.86	15.94 - 77.75
Zooplankton	13C_PCB138	42	50.24 ± 12.56	16.04 - 74.04
Zooplankton	13C_PCB153	42	52.06 ± 13.61	16.85 - 76.93
Zooplankton	13C_PCB180	42	43.96 ± 11.15	14.36 - 62.73
Zooplankton	13C_PCB28	42	44.45 ± 12.37	13.96 - 67.95
Zooplankton	13C_PCB52	42	46.5 ± 14.16	14.24 - 73.54
Zooplankton	13C_aHCH	23	29.39 ± 8.49	10.38 - 40.82
Zooplankton	13C_bHCH	23	30.55 ± 9.79	9.56 - 47.71
Zooplankton	13C_cis-chlordane	23	42.29 ± 12.38	14.99 - 61.76
Zooplankton	13C_gHCH	23	29.75 ± 8.8	10.49 - 41.73
Zooplankton	13C_opDDD	23	48.36 ± 14	17.56 - 70.78
Zooplankton	13C_ppDDE	23	44.88 ± 13.95	14.77 - 67.32
Zooplankton	13C_ppDDT	23	73.48 ± 21.22	27.8 - 110.12
Zooplankton	13C_trans-chlordane	23	41.37 ± 13.47	14.7 - 60.55
Zooplankton	13C_trans-nonachlor	23	41.55 ± 12.4	13.2 - 60.94
Benthos	13C_HCB	34	32.21 ± 7.88	20.19 - 57.36
Benthos	13C_PCB101	34	49.33 ± 13.92	28.34 - 93.31
Benthos	13C_PCB118	34	51.55 ± 14.22	31.19 - 92.31
Benthos	13C_PCB138	34	53.94 ± 15.06	31.54 - 97.62
Benthos	13C_PCB153	34	54.75 ± 15	32.19 - 101.13
Benthos	13C_PCB180	34	49.55 ± 13.85	29.69 - 89.26
Benthos	13C_PCB28	34	42.61 ± 11.74	24.59 - 80.81
Benthos	13C_PCB52	34	44.25 ± 14.09	24.39 - 94.5
Benthos	13C_aHCH	10	32.04 ± 10.68	19.22 - 55.12
Benthos	13C_bHCH	10	35.05 ± 12.25	19.65 - 61.03
Benthos	13C_cis-chlordane	10	49.04 ± 15.13	27.92 - 82.28
Benthos	13C_gHCH	10	33.59 ± 10.77	19.78 - 56.5
Benthos	13C_opDDD	10	53.88 ± 16.11	29.73 - 86.83
Benthos	13C_ppDDE	10	47.21 ± 13.71	26.6 - 75.81
Benthos	13C_ppDDT	10	85.91 ± 22.89	49.13 - 123.12
Benthos	13C_trans-chlordane	10	49.29 ± 15	28.11 - 81.87

Benthos	13C_trans-nonachlor	10	45.6 ± 13.84	25.48 - 75.75
Sculpin	13C_HCB	30	37.62 ± 8.47	17.12 - 49.6
Sculpin	13C_PCB101	30	47.8 ± 10.91	25.33 - 63.57
Sculpin	13C_PCB118	30	49.7 ± 11.55	25.21 - 68.09
Sculpin	13C_PCB138	30	53.82 ± 12.99	26.92 - 73.81
Sculpin	13C_PCB153	30	54.52 ± 13.27	26.93 - 75.69
Sculpin	13C_PCB180	30	50.7 ± 12.75	24.32 - 70.62
Sculpin	13C_PCB28	30	46.08 ± 9.72	24.71 - 59.72
Sculpin	13C_PCB52	30	44.15 ± 9.66	23.87 - 56.97
Lab blank	13C_aHCH	10	36.67 ± 9.08	20.69 - 51.7
Lab blank	13C_bHCH	10	41.71 ± 8.99	31.07 - 56.18
Lab blank	13C_cis-chlordane	10	53.78 ± 10.82	38.11 - 69.35
Lab blank	13C_gHCH	10	39.76 ± 8.48	29.03 - 53.61
Lab blank	13C_opDDD	10	61.73 ± 13.4	45.46 - 82.87
Lab blank	13C_ppDDE	10	55.73 ± 11.63	38.72 - 72.27
Lab blank	13C_ppDDT	10	81.77 ± 25.2	40.79 - 119.57
Lab blank	13C_trans-chlordane	10	54.13 ± 10.69	37.98 - 70.03
Lab blank	13C_trans-nonachlor	10	50.26 ± 11.05	36.16 - 67.05
Lab blank	13C_HCB	17	31.28 ± 14.6	0.38 - 50.3
Lab blank	13C_PCB101	17	62.96 ± 14.46	39.9 - 90.03
Lab blank	13C_PCB118	17	61.38 ± 11.71	42.17 - 82.84
Lab blank	13C_PCB138	17	65.17 ± 10.94	45.31 - 81.91
Lab blank	13C_PCB153	17	66.62 ± 11.66	47.24 - 85.89
Lab blank	13C_PCB180	17	59.8 ± 9.34	42.87 - 74.01
Lab blank	13C_PCB28	17	52.27 ± 14.44	25.32 - 76.4
Lab blank	13C_PCB52	17	57.51 ± 15.73	37.01 - 86.67
SRM	13C_HCB	5	28.92 ± 13.5	11.88 - 41.14
SRM	13C_PCB101	5	41.08 ± 16.87	19.49 - 62.12
SRM	13C_PCB118	5	42.32 ± 17.87	20.4 - 64.65
SRM	13C_PCB138	5	45 ± 18.44	22.49 - 67.98
SRM	13C_PCB153	5	44.87 ± 18.88	22.27 - 68.25
SRM	13C_PCB180	5	41.28 ± 17.33	20.68 - 62.3
SRM	13C_PCB28	5	37.89 ± 15.55	20.95 - 53.57
SRM	13C_PCB52	5	36.55 ± 14.04	18.99 - 51.9
SRM	13C_aHCH	1	17.79 ± na	17.79 - 17.79
SRM	13C_bHCH	1	26.09 ± na	26.09 - 26.09

SRM	13C_cis-chlordane	1	28.81 ± na	28.81 - 28.81
SRM	13C_gHCH	1	21.06 ± na	21.06 - 21.06
SRM	13C_opDDD	1	35.34 ± na	35.34 - 35.34
SRM	13C_ppDDE	1	25.8 ± na	25.8 - 25.8
SRM	13C_ppDDT	1	50.73 ± na	50.73 - 50.73
SRM	13C_trans-chlordane	2	28.07 ± 0	28.07 - 28.07
SRM	13C_trans-nonachlor	1	26.3 ± na	26.3 - 26.3

Table S4. Chiral analysis description

Chiral analysis of α-HCH and *cis*- and *trans*-chlordane in zooplankton samples was performed using a chiralsil-dex column (12.5 m x 0.25 mm x 0.25 µm (Agilent (chrompack), USA) connected in tandem with a TG5-SILMS ((12.5 m x 0.25 mmx 0.25 µm (Thermo Scientific, UK). The chiral stationary phase is: β-cyclodextrin (chiral selector) that is directly bonded to the dimethylpolysiloxane stationary phase. PTV injection of 2 µL was perfomed using previously described conditions for achiral analysis. A carrier gas flow rate of 1.0 ml/min was used together with the following oven program for chromatographic separation: Initial oven temperature was held at 60°C for 1.5 min and increased at 20°C/min to 110°C, followed by a 1°C/min ramp to 210°C. Analytes were analyzed in electron impact mode using and advanced electron impact (AEI) ion source held at 340°C. Ion transitions and collision energies for α-HCH and chlordane isomers are described in the table below.

Table S4. Ion transitions and collision energies for α-HCH and chlordane isomers

Name	Parent ion	Product ion	Collision energy (eV)
**α-HCH (quantification)**	181	145	14
**α-HCH (qualifier)**	281	181	8
**^{13}C-α-HCH (internal standard)**	187	151	12
**Chlordane (quantification)**	373	266	20
**Chlordane (qualifier)**	375	266	20
**^{13}C-Chlordane**	383	276	14

Table S5. Overview of macrozooplankton and zooplankton size fractions analyzed for contaminant concentrations. Community composition of size fractions are illustrated in Figure S1.

Date	Taxon/Size Fraction	Feeding Group	Wet weight (g)
August	> 1000 um	Herbivores	3.26
August	> 1000 um	Herbivores	3.15
August	> 1000 um	Herbivores	3.19
August	> 1000 um	Herbivores	3.5
August	> 1000 um	Herbivores	3.32
August	> 1000 um	Herbivores	3.2
August	> 1000 um	Herbivores	3.31
August	*Beroe cucumis*	Omni/Predators	3.5
August	*Beroe cucumis*	Omni/Predators	10.14
August	*Beroe cucumis*	Omni/Predators	5.09
August	Chaetognatha	Omni/Predators	4.5
August	*Cyanea capillata*	Omni/Predators	4.1
August	*Leptoclinus maculatus*	Omni/Predators	2.35
August	*Mertensia ovum*	Omni/Predators	24.9
August	*Mertensia ovum*	Omni/Predators	10.11
August	*Mertensia ovum*	Omni/Predators	4.33
August	*Thysanoessa* spp.	Omni/Predators	3.44
August	*Thysanoessa* spp.	Omni/Predators	3.48
June	> 1000 um	Herbivores	3.38
June	> 1000 um	Herbivores	3.38
June	> 1000 um	Herbivores	3.11
June	> 1000 um	Herbivores	3.23
June	> 1000 um	Herbivores	3.31
June	> 1000 um	Herbivores	3.18
June	> 1000 um	Herbivores	3.51
June	> 1000 um	Herbivores	2.94
June	> 1000 um	Herbivores	3.18
June	> 1000 um	Herbivores	4.04
June	> 1000 um	Herbivores	3.57
June	> 1000 um	Herbivores	3.3
June	> 1000 um	Herbivores	3.18

June	500-1000 um	Herbivores	3.43
June	500-1000 um	Herbivores	3.2
May	> 1000 um	Herbivores	3.48
May	> 1000 um	Herbivores	3.13
May	> 1000 um	Herbivores	3.04
May	> 1000 um	Herbivores	3.59
May	> 1000 um	Herbivores	3.69
May	500-1000 um	Herbivores	3.71
May	500-1000 um	Herbivores	3.53
May	500-1000 um	Herbivores	3.05
May	Chaetognatha	Omni/Predators	5.23
May	*Leptoclinus maculatus*	Omni/Predators	2.64
May	*Thysanoessa* spp.	Omni/Predators	3.16

Table S6. Overview of zoobenthic taxa analyzed for contaminant concentrations.

Fjord	Taxon	Feeding Group	wet weight (g)
Adventfjorden	Ascidiacea	Filter feeder	3.13
Adventfjorden	Ascidiacea	Filter feeder	3.15
Adventfjorden	*Hyas* sp.	Scavenger	3.02
Adventfjorden	*Hyas* sp.	Scavenger	3.13
Adventfjorden	*Mya arenaria*	Filter feeder	4.88
Billefjorden	Ascidiacea	Filter feeder	3.28
Billefjorden	*Ciliatocardium cilliatum*	Filter feeder	1.86
Billefjorden	*Hyas* sp.	Scavenger	2.76
Billefjorden	*Leptasterias* sp.	Scavenger	3.22
Billefjorden	*Serripes groenlandicus*	Filter feeder	3.12
Isfjorden	*Astarte* sp.	Filter feeder	2.81
Isfjorden	*Maldane sarsi*	Deposit feeder	3.1
Isfjorden	*Maldane sarsi*	Deposit feeder	1.87
Isfjorden	*Pandalus borealis*	Predator	3.21
Isfjorden	*Pandalus borealis*	Predator	3.27
Isfjorden	*Pandalus borealis*	Predator	3.14
Isfjorden	*Sabinea septemcarinata*	Predator	3.63
Isfjorden	*Sabinea septemcarinata*	Predator	3.8
Isfjorden	*Sabinea septemcarinata*	Predator	3.7
Tempelfjorden	Ascidiacea	Filter feeder	3.03
Tempelfjorden	*Astarte* sp.	Filter feeder	4.35
Tempelfjorden	*Hyas* sp.	Scavenger	3.09
Tempelfjorden	*Hyas* sp.	Scavenger	2.21
Tempelfjorden	*Macoma calcarea*	Deposit feeder	1.93
Tempelfjorden	Nephtyidae	Predator	3.04
Tempelfjorden	*Sabinea septemcarinata*	Predator	3.27

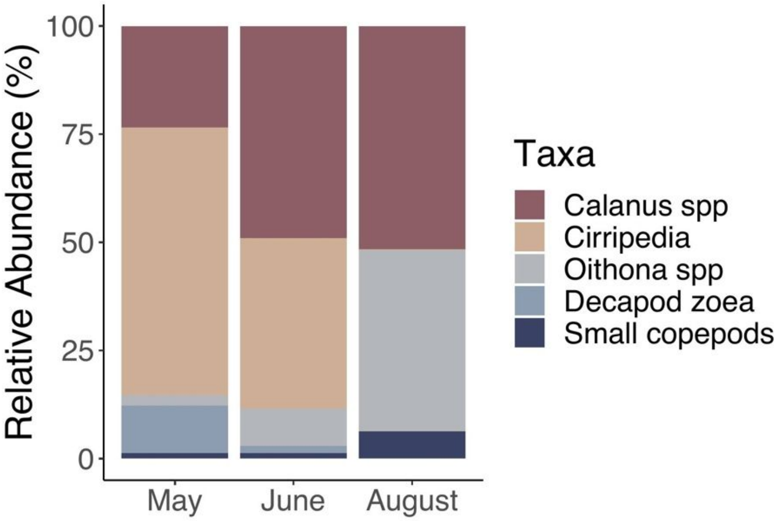

Figure S1. Relative abundance of main zooplankton taxa in size fractionated samples within each month.

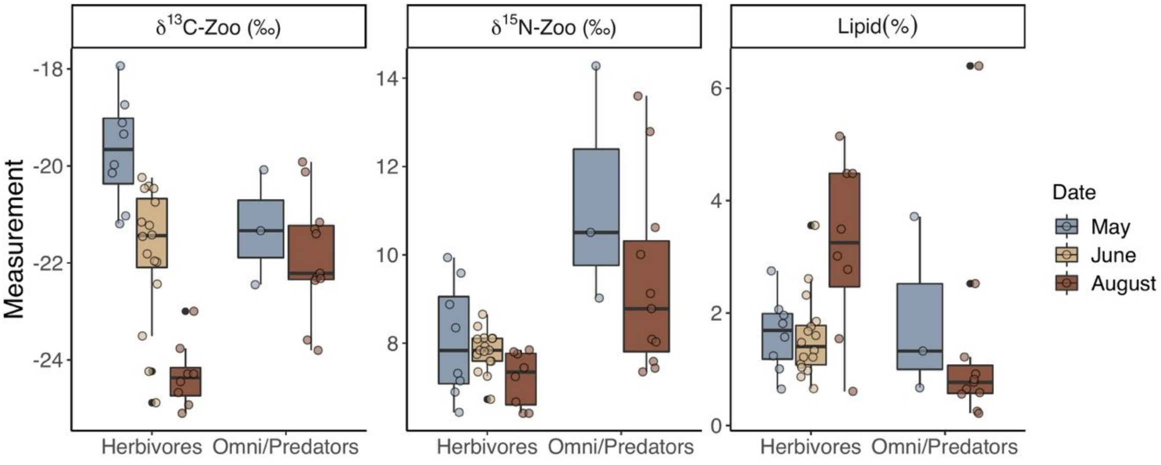

Figure S2. $\delta^{13}C$, $\delta^{15}N$ and lipid content in zooplankton feeding groups within each month.

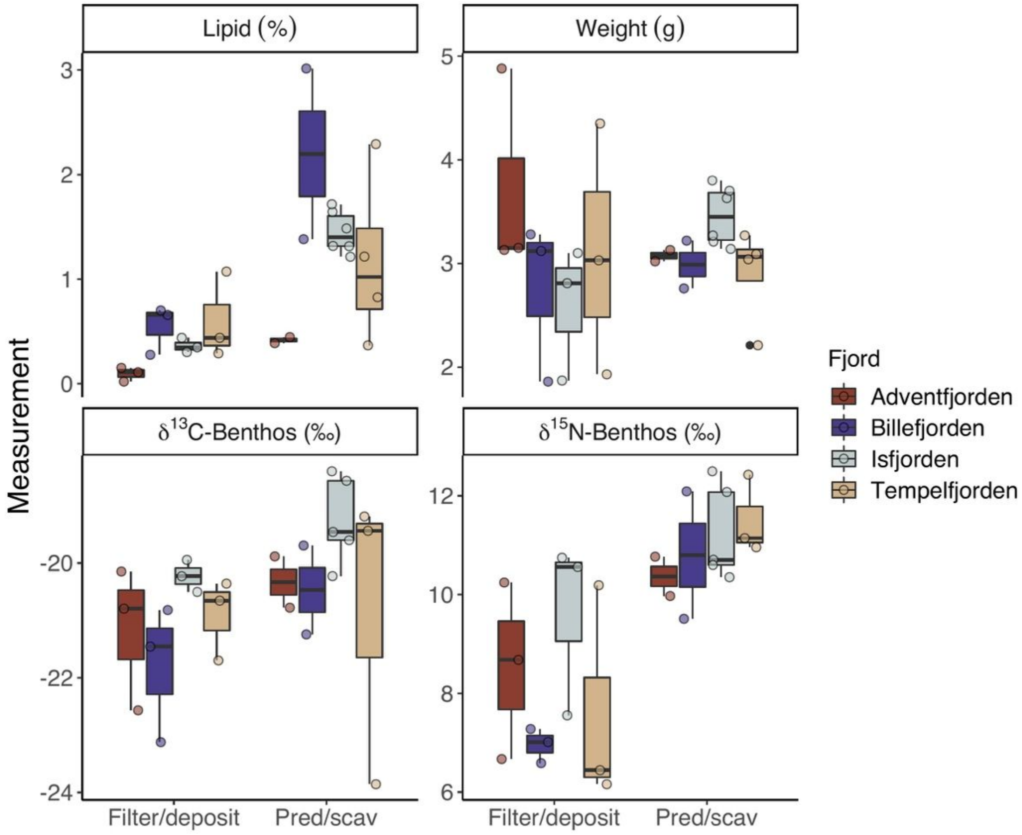

Figure S3. Lipid content, wet weight, δ^{13}C and δ^{15}N of benthic invertebrates (filter/deposit feeders and predator/scavengers) among sampled fjords.

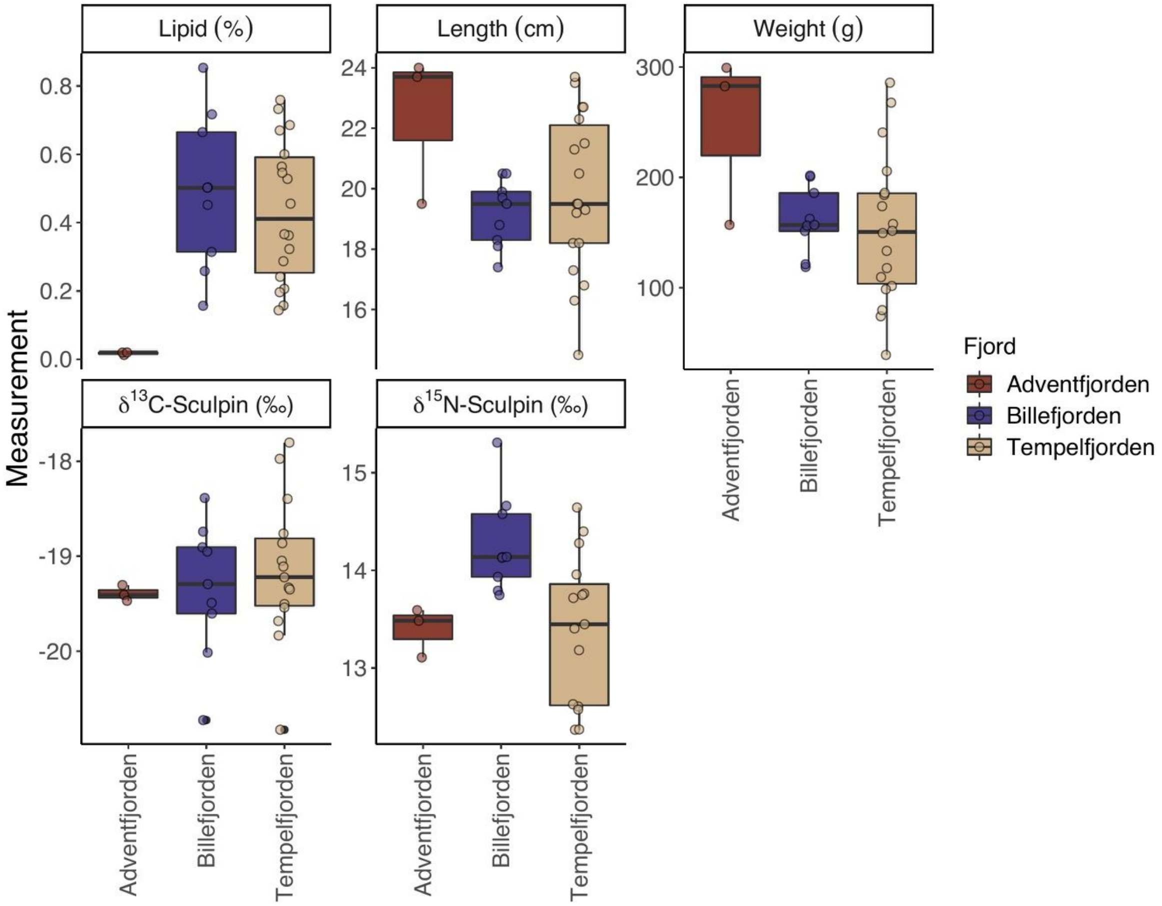

Figure S4. Lipid content, fish length, wet weight, δ^{13}C and δ^{15}N of sculpin among sampled fjords.

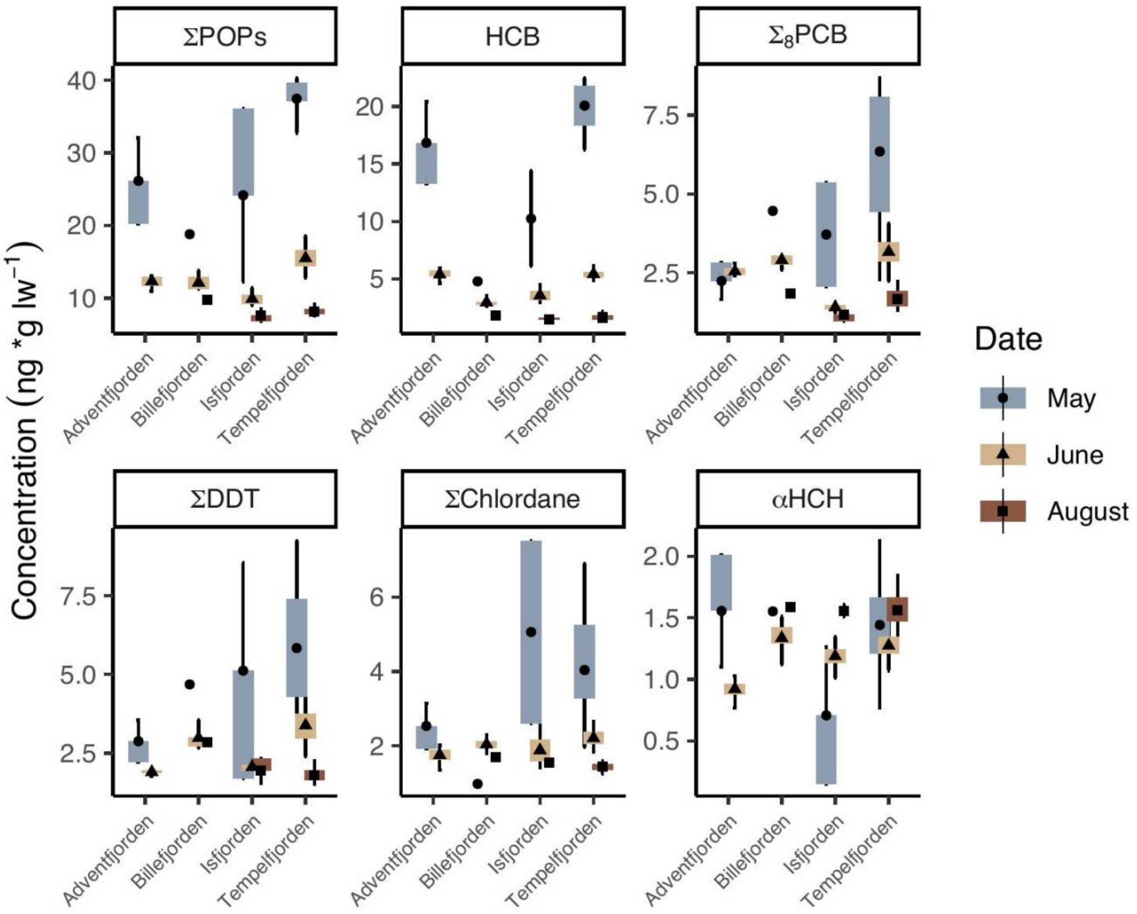

Figure S5. Spatial patterns in herbivorous zooplankton contaminant concentrations by fjord within each month.

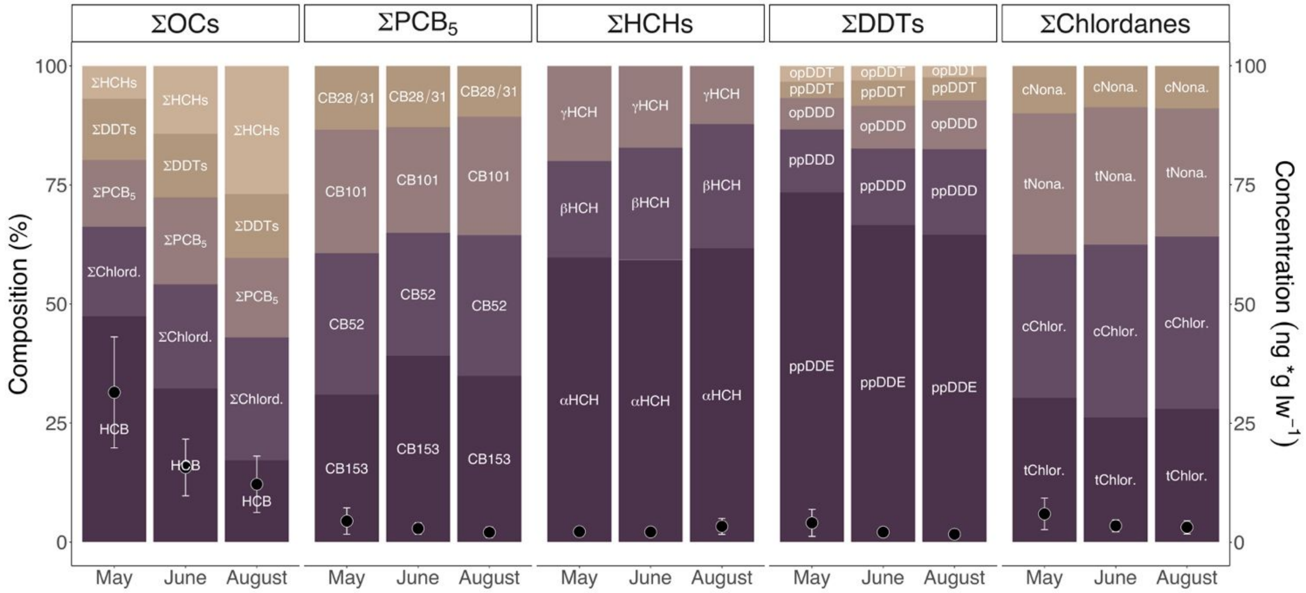

Figure S6. Compositional contaminant profiles for herbivorous zooplankton.

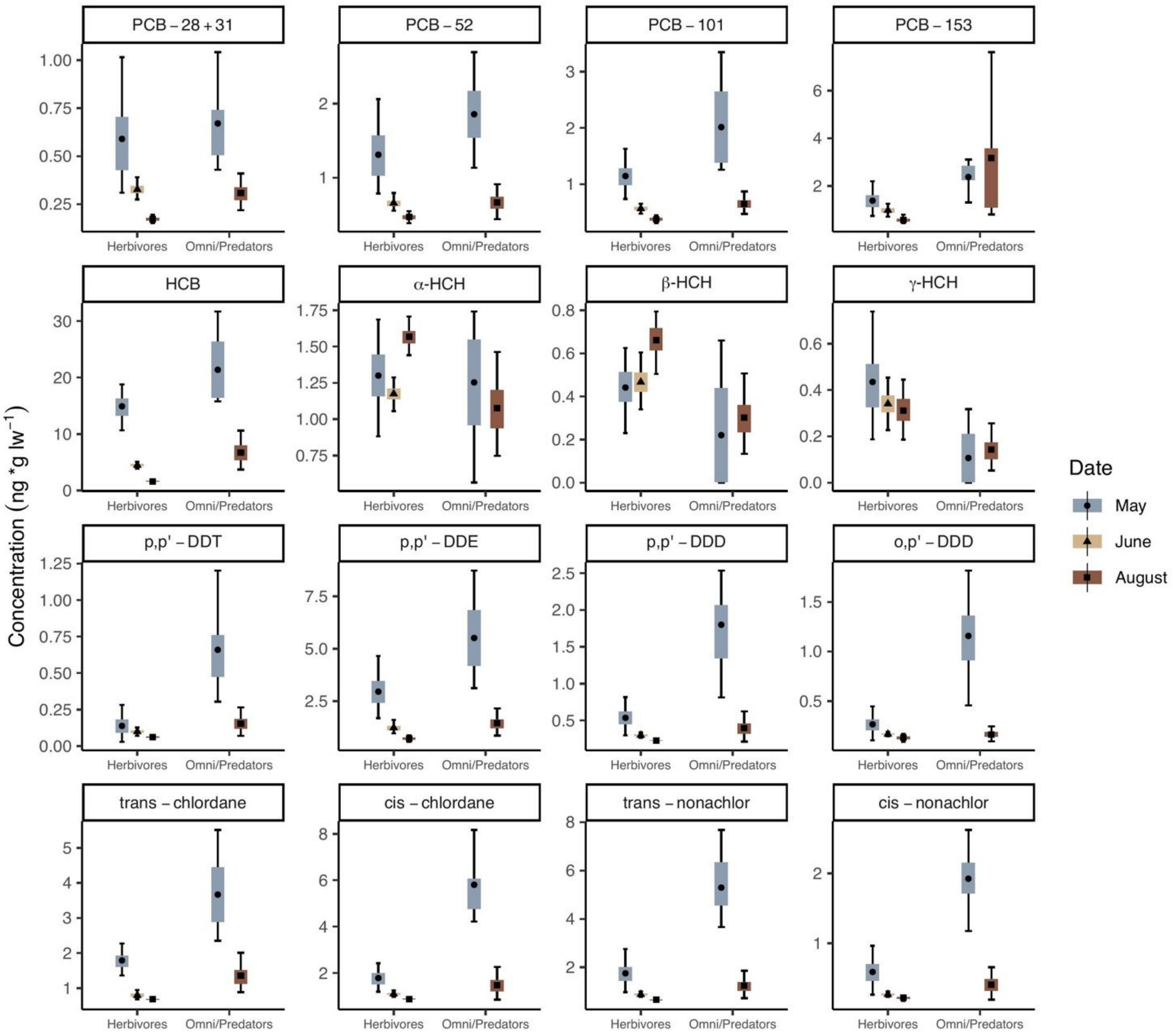

Figure S7. Congener- and isomer-specific contaminant concentrations in zooplankton on the lipid weight basis in each month.

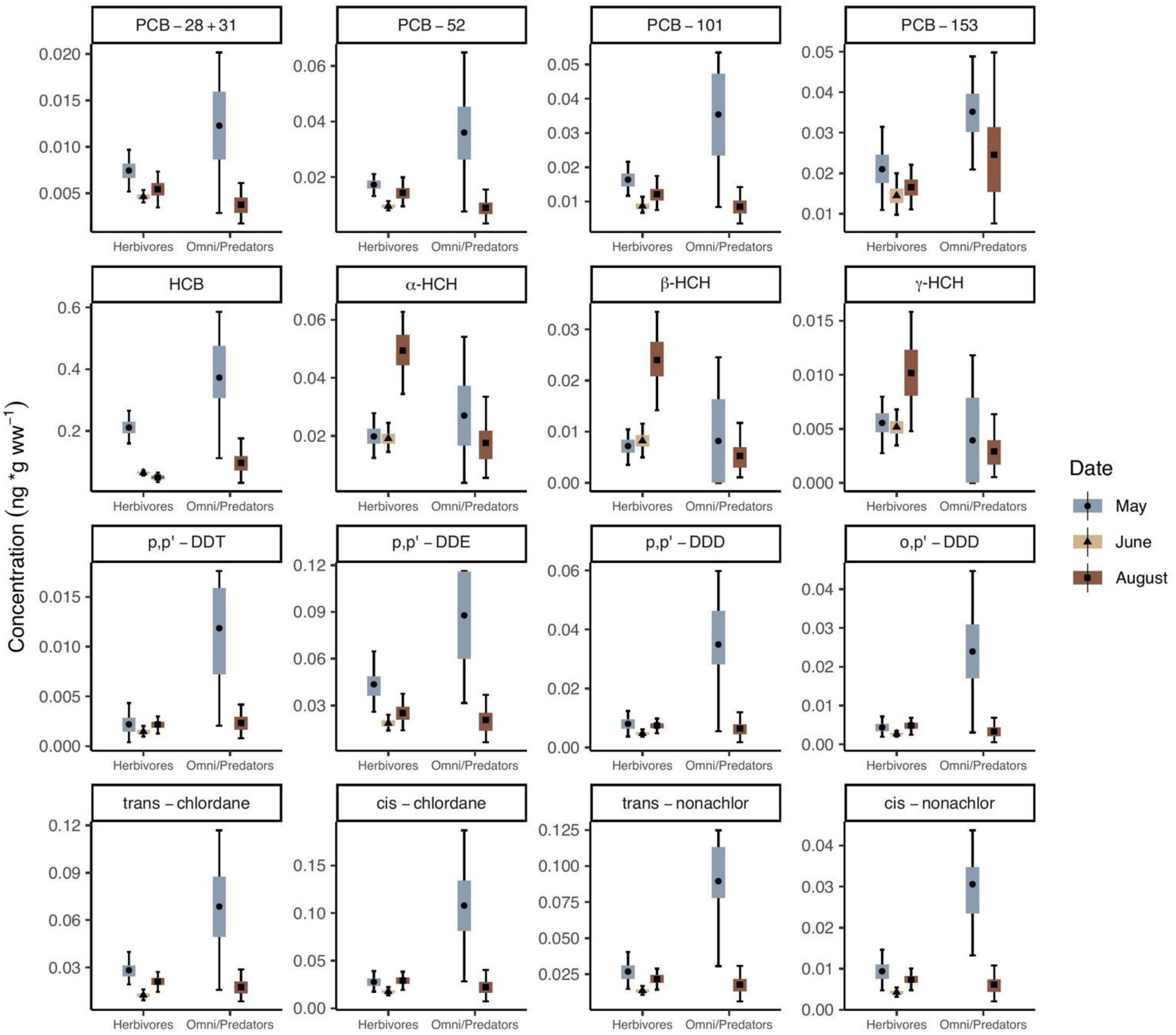

Figure S8. Congener- and isomer-specific contaminant concentrations in zooplankton on the wet-weight basis in each month.

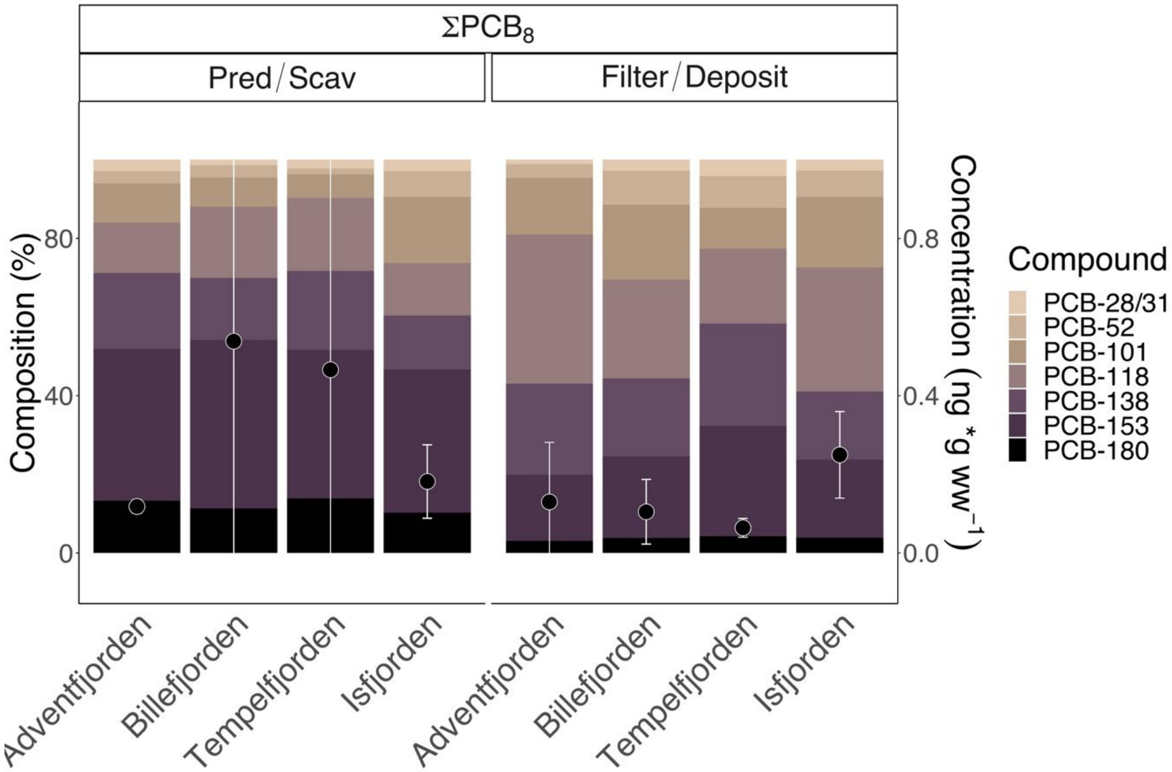

Figure S9. Composition of ∑PCB8 in benthos from each fjord grouped by feeding habit: predators and scavengers vs. filter- and deposit-feeders.

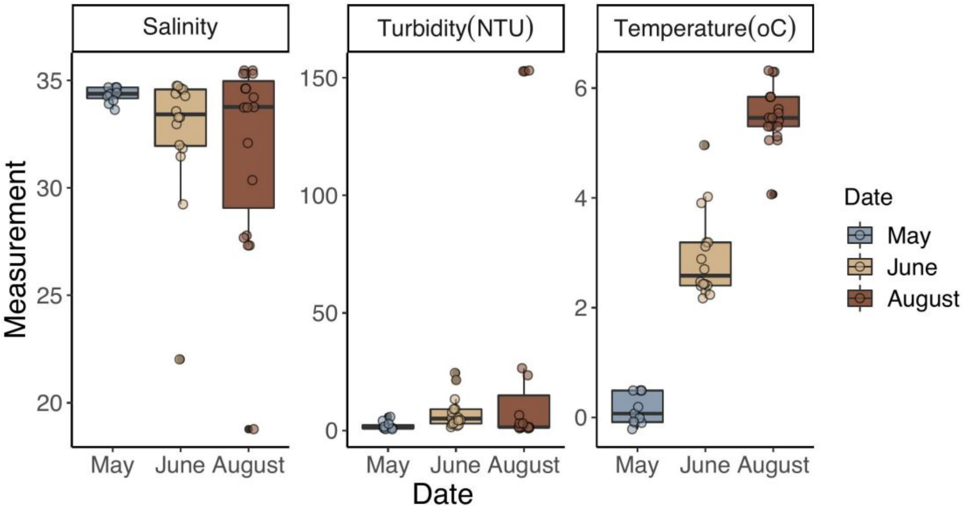

Figure S10. Overview of environmental variables used in RDA analysis and variance partitioning. For more information, see McGovern et al. (2020).

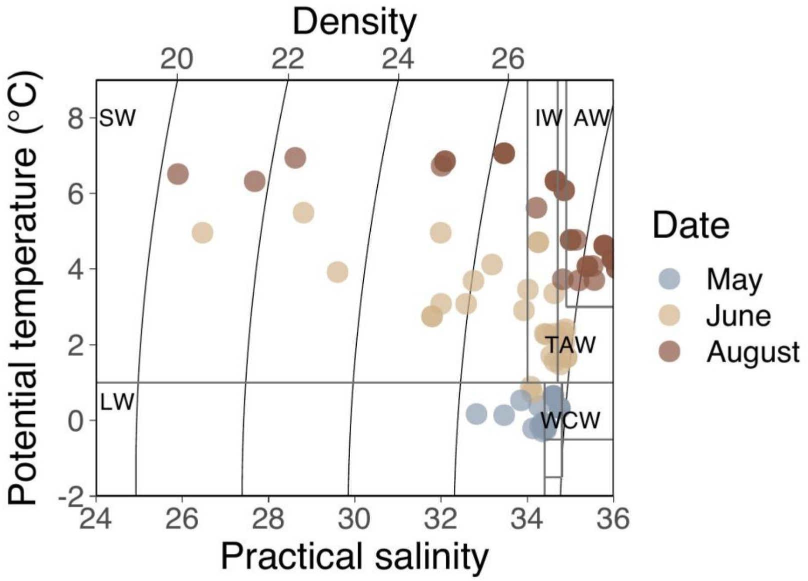

Figure S11. Temperature-Salinity diagram of water samples collected alongside zooplankton samples. Discrete water samples from surface and 15m are both included. This diagram was made using the PlotSvalbard R package (Vihtakari, 2019) using water mass determinations based on Nilsen et al. (2008). SW = surface water, IW= intermediate water, AW= Atlantic water, TAW = transformed Atlantic water, ArW = Arctic water, WCW = winter cooled water and LW = local water.

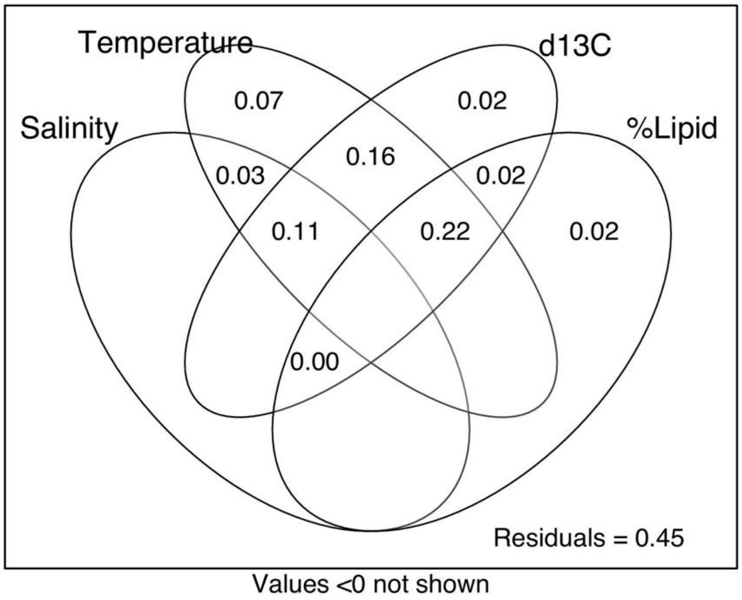

Figure S12. Results of variance partitioning based on log transformed contaminant concentrations in herbivorous zooplankton.

References

McGovern M, Pavlov A, Deininger A, Granskog M, Leu E, Søreide JE, Poste AE (2020). Terrestrial Inputs Drive Seasonality in Nutrient and Organic Matter Biogeochemistry in a High-Arctic Fjord System (Isfjorden, Svalbard). Frontiers Marine Science. doi: 10.3389/fmars.2020.542563

Nilsen, F., Cottier, F., Skogseth, R., and Mattsson, S. (2008). Fjord-shelf exchanges controlled by ice and brine production: the interannual variation of Atlantic Water in Isfjorden, Svalbard. Contin. Shelf Res. 28, 1838–1853. doi: 10.1016/j.csr.2008.04.015

Vihtakari, M. (2019). PlotSvalbard: PlotSvalbard – Plot Research Data From Svalbard on Maps. Rpackage version 0.8.5.
